# Quantification of Partial Oxidation of Magnetite to Maghemite in Aqueous Solution Using XRD

**DOI:** 10.1021/acsomega.6c01180

**Published:** 2026-10-15

**Authors:** Morgane Desmau, Thaïs Couasnon, Roberts Blukis, Jeffrey Paulo H. Perez, Liane G. Benning

**Affiliations:** † Deutsches Elektronen-Synchrotron DESY, Notkestr. 85, Hamburg 22607, Germany; ‡ GFZ Helmholtz Center for Geosciences, Telegrafenberg, Potsdam 14473, Germany; § Leibniz-Institut für Kristallzüchtung, Max-Born Str. 2, Berlin 12489, Germany; ∥ Department of Earth Sciences, Freie Universität Berlin, Berlin 12249, Germany

## Abstract

In aqueous solutions, magnetite (Fe_3_O_4_) nanoparticles (NPs) easily oxidize into a material with crystalline characteristics in between magnetite and maghemite (γ-Fe_2_O_3_), inevitably modifying their properties. The routine quantification and characterization of this transformation were previously hampered by the structural similarity of magnetite and maghemite. Here, we compared X-ray Absorption Fine Structure (XAFS) characterization to laboratory-based X-ray Diffraction (XRD) pattern analysis, such as Rietveld refinement and peak position shifts of the (311), (400), (511), and (440) reflections, to determine if XRD can be used to determine magnetite oxidation by itself. We analyzed the transformation extent of nanoparticles (10 nm) reacting under different concentrations of oxidizing agents such as O_2_ and H_2_O_2_. We showed that XRD methods—Rietveld refinements and single peak, such as (511)-based—can within error reproduce XAFS results. Our results document a more accessible characterization method for the quantification of magnetite NPs partial oxidation in solution.

## Graphical abstract

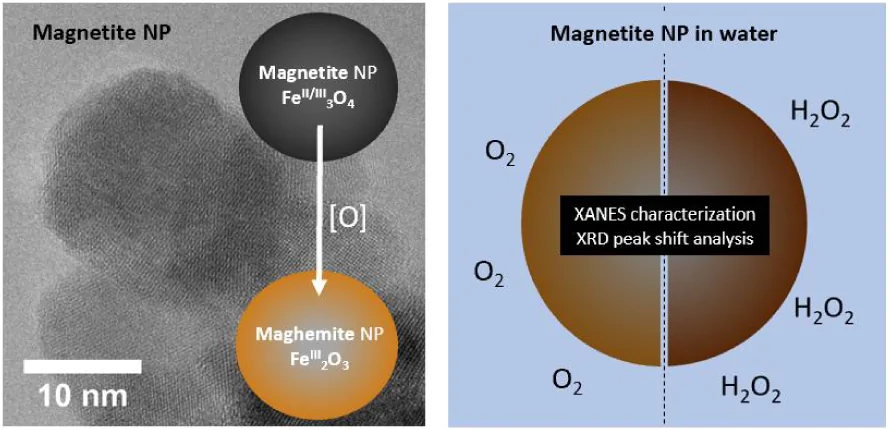

## 1 Introduction

Nanoparticulate magnetite has been shown to be a good candidate for metal­(loid)­s and organic contaminants, including per- and polyfluoroalkyl substances (PFAS), remediation from contaminated groundwaters and soils due to its high surface area, redox activity, and superparamagnetic properties.^1–6^ Such processes often occur in aqueous solutions where magnetite nanoparticles (NPs) may easily oxidize, forming a phase with crystalline characteristics between magnetite and maghemite (γ-Fe_2_O_3_).^7^ As the redox reactivity of magnetite and maghemite is different,^8,9^ it is essential to be able to understand and characterize the partial or complete oxidation of magnetite to maghemite in the presence of naturally occurring oxidizing agents and thus predict the changes in properties of magnetic NP in natural and contaminated environments.^10^

The two iron oxides have isomorphous cubic spinel crystal structures in which the metallic cations occupy tetrahedral and octahedral interstices. In the inverse spinel structure of magnetite, all the tetrahedral sites are exclusively occupied by Fe^3+^ while Fe^2+^ and Fe^3+^ evenly fill the octahedral sites. The stoichiometry of magnetite (R) NPs, which is one of the most important factors controlling their reactivity, can be defined as R = Fe^2+^/Fe^3+^. Magnetite oxidizes to maghemite by converting all Fe^2+^ into Fe^3+^, with R going from 0.5 to 0, leaving iron vacancies in the crystal lattice to balance the excess positive charge.^11,12^ The two minerals have almost identical compositions, crystalline structures, and comparable unit cell edge lengths (a_magnetite_ = 8.39/8.40 Å ^13–15^ and a_maghemite_ = 8.35 Å, respectively^7^) that increase with the Fe^2+^/Fe^3+^ ratio.^16^ Their identification and, in particular, the proportion of magnetite oxidized into maghemite is usually hindered by the difficulty in quantifying the magnetite-maghemite ratio content.^8^

Many laboratory studies attempted to measure the magnetite-maghemite ratio in mineral mixtures or to identify the nonstoichiometry of magnetite NP powders through one or a combination of the following techniques: Mössbauer spectroscopy, Raman spectroscopy, infrared spectroscopy, X-ray Photoelectron Spectroscopy (XPS), X-ray Absorption Fine Structure (XAFS), transmission electron microscopy (TEM) with selected area electron diffraction, wet chemistry, and X-ray magnetic circular dichroism (XMCD) analysis.^13,17–22^ Among those, Mössbauer spectroscopy is widely used to characterize iron oxides since they present different magnetic hyperfine patterns,^21^ which can be used to differentiate magnetite from maghemite for NPs with diameters above 15 nm at room temperature. For smaller NPs, low temperature (liquid He temperature) or application of an external magnetic field is needed.^21^ On the other hand, Raman spectroscopy is a rapid and efficient screening tool when the pool of samples to analyze is large. However, accurate quantification of the magnetite-maghemite ratios is not possible.^23^ Wet chemistry based on complete dissolution and Fe^2+^/Fe­(total) determination is widely used to estimate Fe^2+^/Fe^3+^ ratios.^11^ However, in complex systems containing multiple Fe-bearing phases, organic ligands, and dissolved redox-active species, complementary spectroscopic or magnetic techniques are often employed for stoichiometry determination.^22,24^ The combination of several techniques, such as XPS, Raman spectroscopy, and/or Mössbauer spectroscopy, has been successfully used to characterize magnetite and maghemite NPs that were synthesized through different methods^17^ or in different chemical environments.^25^ Synchrotron-related techniques can also be used to investigate the magnetite-to-maghemite ratio: XMCD, for example, enables one to distinguish the tetrahedral (Td) and octahedral (Oh) iron and their oxidation state in magnetic compounds.^13^ XAFS has also been widely used to study iron oxide minerals in complex environments.^26–28^ In particular, XAFS spectra can be used to distinguish between magnetite, maghemite, and partially oxidized magnetite, notably through structural (coordination, metal–ligand distances) and electronic (oxidation state, spin state) information.^29–33^ Despite numerous advantages, such as minimal sample preparation and high-throughput options, XAFS and XMCD measurements require access to synchrotron facilities, limiting their use as a routine technique. While all these techniques provided insightful information regarding the evolution of magnetite stoichiometry in solution, quantitative determination of magnetite-maghemite proportions remains challenging because partially oxidized magnetite do not form discrete intermediate phases but rather a continuum of nonstoichiometric compositions.^8,34^

Other studies have used X-ray Diffraction (XRD) combined with various techniques to investigate structural distortions, vacancy ordering, and nonuniform unit cells.^35,36^ They showed that local disorder and variable unit cells within individual particles require advanced modeling (e.g., PDF analysis) and complementary techniques to interpret XRD results. Likewise, Volenik and collaborators (1975),^37^ Yang and collaborators (2004),^38^ or Schmidbauer and collaborators (2006)^39^ emphasized cross-validation with spectroscopic and magnetic methods to determine Fe^2+^/Fe^3+^ ratios and phase fractions. These studies demonstrated that XRD alone cannot accurately quantify the extent of magnetite oxidation; rather, they collectively highlight that accurate characterization of oxidation requires complementary techniques and advanced structural analysis. However, other studies suggested that X-ray Diffraction (XRD) may be a powerful routine tool to investigate the relative abundance of the magnetite/maghemite ratio or the extent of oxidation in nonstoichiometric magnetite in a sample.^22,24^ The stoichiometry of the magnetite NPs can be linked to their XRD patterns:^13^ an oxidation of the magnetite NPs leads to nonstoichiometric magnetite with smaller cell parameters.^11,13,16^ For example, Gorski and Scherer (2010)^11^ reported shifts in XRD peak position associated with magnetite oxidation. They investigated synthetic magnetite nanoparticles spanning a wide range of stoichiometries and used whole-pattern pseudo-Voigt refinement of pXRD data to determine lattice parameter.^11^ They compared the obtained lattice parameter values to the stoichiometry values of the synthesized magnetite NPs with Mössbauer spectroscopy measurements and acidic dissolution coupled with the 1,10-phenanthroline method.^11^ They demonstrated a strong linear relationship between lattice parameter and magnetite stoichiometry, showing that XRD can be used to estimate oxidation state. However, their study focused on the determination of stoichiometry through refined lattice parameters rather than on establishing a direct calibration based on individual diffraction peak shifts. In addition to the relation between R and cell parameters, peak position shifts for some XRD peaks (i.e., the 511 or 311 peaks) have also been documented before. Keefer and Shive (1981), also using XRD, reported that an oxidation parameter based on the position of the (511) reflection allows quantification of titanomagnetites and titanomaghemite, linking the unit cell length with the oxidation extent of magnetite, without any independent analytical comparison.^40^ Hounslow and Maher in 1999 ^41^ adapted the methodology described by Keefer and Shive in 1981 ^40^ to the (311) peak, while the original work was based on the (511) peak analysis. Finally, Jørgensen et al. (2007)^42^ followed changes in the (311) peak position for maghemites synthesized by thermal decomposition of an amorphous precursor, but they did not provide any independent validation of their results. These studies revealed the great potential of XRD pattern analysis as a promising technique to characterize and identify the transformation extent in magnetite, maghemite, and partially oxidized magnetite NPs. However, so far, no study has demonstrated that such XRD methods alone can quantify the relative oxidation of magnetite without complementary techniques.

We propose here that changes in the (311), (400), (511), and (440) Bragg reflections in magnetite XRD patterns, as well as lattice parameters and Fe occupancies based on Rietveld refinement of these patterns, can be robustly used to independently quantify the relative oxidation of magnetite NPs from a well-characterized initial NP state. To test this hypothesis, magnetite NPs were synthesized, and oxidation experiments in aqueous media at variable conditions were conducted. Parameters playing a crucial role in natural environments, such as O_2_ and H_2_O_2_ (used as a reactive oxygen species proxy) concentrations, which were previously investigated,^11–13,43^ were varied to test the robustness of the XRD analysis for environmentally relevant applications. To assess the accuracy of the XRD data evaluation and to provide complementary evidence for the oxidation processes, we employed X-ray absorption fine structure (XAFS).

With our combined XRD and XAFS approach, we deliver deeper insights into an approach that allows the transformation extent and mechanisms involved in the particle transformation of magnetite to be determined with accuracy, thus allowing a better predictability of magnetite-maghemite particle properties in different environments.

## 2 Experimental Section

### 2.1 Nanoparticle Synthesis

Magnetite NP synthesis was performed in an anaerobic chamber (COY vinyl anaerobic chamber, <2 ppm of O_2_) by aqueous coprecipitation of stoichiometric amounts of ferric (FeCl_3_·6H_2_O, Carl Roth GmbH & Co.) and ferrous iron (FeCl_2_·4H_2_O, Sigma-Aldrich) chlorides dissolved in O_2_-free ultrapure water basified to pH 12 by 1 M NaOH. The pH electrode was calibrated with NIST-certified buffers (4, 7, and 10) and was connected to a DrDAQ data logger. Following precipitation, the magnetite NPs were vacuum-dried on a 0.2-μm cellulose filter, and any excess salts were washed off the filter with O_2_-free ultrapure water. Maghemite NPs were synthesized by heating the initial magnetite NPs at 200 °C overnight in ambient oxic conditions.

### 2.2 Oxidation of Magnetite NPs in Aqueous Suspensions

All experiments were performed at 25 °C. O_2_-free ultrapure water (18.2 MΩ·cm^–1^) was produced by bubbling N_2_ gas while boiling for 4 h. After cooling, the water was transferred into the anaerobic chamber. In addition, all experiments were performed in crimped vials to consistently maintain O_2_-free conditions throughout all reactions. For the induced oxidation experiments, the O_2_-free water was pH adjusted to 8.3 and reacted with the magnetite NPs (1.54 mg mL^–1^) at one of five concentrations of O_2_ ranging from anaerobic to air-saturated water (6·10^–6^ M, 1.37·10^–4^ M, 2.30·10^–4^ M, 2.60·10^–4^ M, and 2.66·10^–4^ M) or four concentrations of H_2_O_2_ (10^–9^ M, 10^–7^ M, 10^–5^ M, and 10^–3^ M). Following the mixing of the NPs with the reacting solutions, the crimped vials were sealed, the initial pH of the suspensions was measured, and the contents were kept at room temperature on an orbital shaker at 170 rpm (IKA shaker KS 4000). After 7 days of reaction, the final pH was remeasured in every NPs suspension under anaerobic conditions in the anaerobic chamber. No significant change in pH (i.e., <0.1) was observed between the initial and final pH values. The pH values presented in the figures are the final pH. Subsequently, the NPs were vacuum-dried on a 0.2-μm cellulose filter and washed on the filter with O_2_-free ultrapure water. In order to gain information on the mechanism of the magnetite transformation in these experiments, initial dissolved O_2_ contained in each closed vial was monitored prior to magnetite addition. It was compared to the concentration obtained at the end of the 7-day exposure in blank experiments (i.e., without magnetite NPs) and in the presence of magnetite nanoparticles.

### 2.3 Aqueous O_2_ Monitoring

Dissolved oxygen was monitored using a Unisense x-5 UniAmp multichannel amplifier (Aarhus, Denmark) equipped with a temperature sensor and a standard glass needle sensor (OX-NP) used to pierce the hermetically sealed crimped vials. A two-point calibration was performed prior to each measurement using the Unisense logger software and a standard O_2_-free solution from a Unisense calibration kit and an O_2_-saturated solution made by bubbling air through water for 10 min in a Unisense calibration chamber.

### 2.4 X-ray Diffraction (XRD) Acquisition and Analysis

X-ray diffraction patterns were acquired with a STOE STADI P instrument equipped with an Ag Kα source (λ = 0.55941 Å), a curved Ge (111) monochromator, and two DECTRIS MYTHEN 2 detectors in a Debye–Scherrer geometry. Measurements were performed over the 2θ range of 0–77° with an angular resolution of 0.015°. The average counting time per sample was 4 h (about 2 h per data point). To accurately quantify and compare the lattice parameters between samples, the dried samples were mixed with an internal Si standard prior to filling into 0.5 mm diameter glass capillaries (Hilgenberg 4007405) and sealed with wax (Hawksley Cristaseal) to maintain the O_2_-free conditions with all manipulations performed inside an anaerobic chamber.

Data analysis was performed by Rietveld refinements using the GSAS-II software.^44^ Magnetiteand maghemite (*Fd*3̅m),^45^ maghemite (*P*4_3_2_1_2),^46^ maghemite (*P*4̅3*m*),^47^ maghemite (*P*4_1_32),^47^ maghemite (*P*4_3_32),^48^ and silicon were modeled using *CIF* files available in the AMCSD database. Out of these structural models, maghemite (*P*4_3_32) was chosen as the most appropriate, as it is the simplest model that described all significant features of the observed XRD patterns. During the refinement, unit, grain size, microstrain, thermal displacement, atomic positions (tetragonal maghemite only), and Fe atom occupancy parameters were optimized. In addition, the background intensity was approximated by Chebyshev polynomials. Sample absorption was estimated based on sample size and density. The instrument function parameters were obtained by empirically fitting a NIST Si standard.

Another method to determine the extent of magnetite oxidation from XRD patterns consisted of analyzing the shift of the position of 4 different XRD reflections: (311), (400), (511), and (440). The positions of these peaks in magnetite were quantified using Igor Pro v8.04. The background corresponding to an empty capillary was subtracted from all XRD patterns. For each reflection, the as-synthesized magnetite and the maghemite standard samples served as experimental reference materials, and the positions of the sample reflections allowed us to model the magnetite-to-maghemite ratio in each sample.

### 2.5 TEM Data Acquisition

Particle sizes and shapes were investigated by transmission electron microscopy (TEM) using an FEI Tecnai G2 F20 X-Twin TEM instrument equipped with a field-emission gun electron source. For each sample, a few milligrams of sample were dispersed in 1 mL of acetone, ultrasonicated for 2 min, and drop-cast deposited onto holey carbon copper TEM grids. Images were acquired at an electron voltage of 200 kV as energy-filtered images on a Gatan GIF Tridiem detector. As magnetite particles aggregate on the TEM grid, images were also collected in TEM dark-field mode in order to ensure the correct observation of particle sizes. Particle size distributions were evaluated by measuring between 130 and 215 particles per sample.

### 2.6 XAFS Experiments

XAFS sample analyses were conducted at the Fe K-edge (7112 eV) on the P65 undulator beamline of the Deutsches Elektronen-Synchrotron (HASYLAB/DESY PETRA III, Hamburg, Germany),^49^ operated at 6.0 GeV with a 100 mA current in multi-bunch mode. The incoming photon flux energy was modulated with a Si(111) double crystal monochromator, with an energy resolution of ∼0.7 eV at the Fe K-edge and a beam size of 0.3 × 1.5 mm^2^. The effective suppression of higher harmonics was achieved using Si-plane mirrors. The data were collected from −150 eV below to +1000 eV above the edge with a scan energy increment of ∼0.6 eV in continuous mode. The time for each spectrum was 240 s, and an average of 5 scans was used for the analysis of each sample. Spectra of reference compounds and samples were acquired in transmission mode, concomitantly with the spectrum of an Fe foil for energy calibration and alignment. The first inflection point in the first derivative of the adsorption threshold of the Fe foil was calibrated at 7112 eV. Incident and transmitted X-ray intensities were recorded using ion chambers with a path length of 5 cm. The ion chambers were filled with a gas mixture of N_2_, Ar, and Kr to approximately obtain 15%, 50%, and 100% absorption for the incident beam I_0_, the transmitted beam I_T_, and the beam transmitted through the reference foil I_T2_, respectively.

Inside the anaerobic chamber, all experimental samples as well as the synthesized reference compounds (magnetite and maghemite) were carefully mixed with cellulose (2.5% iron), pressed into ∼1 mm thick and 13 mm diameter pellets with a high-pressure press, and sealed with Kapton tape to prevent oxidation. The prepared pellets were transferred to DESY inside a sealed anaerobic container and stored inside a glovebox before being mounted in the station’s Oxford Instruments liquid helium flow cryostat. The cryostat was used to maintain the samples and reference compounds at 20 K and at 10^–6^ mbar to avoid oxidation or photoreduction under the beam during XAFS measurement.

Fe K-edge XAS spectra were calibrated, averaged, normalized, analyzed, fitted, and plotted using a series of in-house notebooks written in Python using the Larch^50^ and Araucaria libraries,^51^ NumPy,^52^ SciPy,^53^ and Matplotlib.^54^

For Fe, the oxidation state can be represented by the centroid position of the XANES (X-ray Absorption Near Edge Structure) pre-edge peak.^27^ However, given the monochromator resolution and the step size used to measure the spectra (∼0.6 eV), the XANES pre-edge peaks are poorly resolved and were not used for quantitative analysis in this study. In the absence of the pre-edge peak quantitative values, the energy threshold E_0_ is a good indicator of the oxidation state. The usual way to determine the threshold energy E_0_ is the maximum of the first derivative; however, this does not allow one to quantitatively determine the oxidation states of Fe oxides, specifically in the case of magnetite and maghemite.^31^ Thus, the energy at the half-maximum position in the XANES spectrum was used to determine E_0_ in this study.^51^

E_0_ was measured for various iron oxides with different Fe oxidation states: wüstite (FeO) as a pure Fe^2+^ oxide (provided graciously by Anke Neumann), maghemite,^55^ another Fe^3+^-based iron oxide (ferrihydrite),^56^ and mixtures with different wüstite/maghemite ratios that represent a Fe^2+^/Fe^3+^ ratio (25/75, 45/55, and 79/21). The XAFS spectra of all references used are plotted in ESI-Figure 1.

XANES spectra were used to quantify the transformation of the initial magnetite, characterized as “as-synthesized” in the rest of the manuscript, into mixed phases using linear combination fitting (LCF). LCF analyses were performed from −20 to +70 eV below and above the Fe K-edge (7112 eV). The chi-squared metric (χ^2^) was used to estimate the goodness of the LCF analysis. To include a reference in the fit, the amount of the reference has to be larger than 10% and improve the quality of the fit by 20%. This LCF methodology has an estimated uncertainty of 15%.^57^ The uncertainties in the percentage of the distribution obtained by LCF for each fit were calculated using the Imfit library.^58^ Different iron oxides, including, for example, hematite and goethite, have been tested in the LCF procedure, but none produced satisfactory results, indicating that in this study, initial magnetite is oxidizing to maghemite.

The experimental Extended X-ray Absorption Fine Structure (EXAFS) spectra were fitted to the theoretical scattering paths computed by the FEFF6l computer code,^59,60^ using the Levenberg–Marquardt nonlinear least-squares fitting (LSF) algorithm implemented in Larch. The r-factor was used to estimate the goodness of the fit, and only results with r-values below 0.02 were considered valid. The crystal structures of Fe_3_O_4_ ^61^ and γ-Fe_2_O_3_ ^47^ were used as models for magnetite and maghemite, respectively. The amplitude reduction factor (S_0_^2^) was fixed at 0.79. This value was obtained by merging the S_0_^2^ value of pure magnetite and maghemite with this parameter adjusted to yield the expected number of first nearest neighbors for the model compounds. In the fitting procedure, the EXAFS function was k^2^-weighted and fitted using a Kaiser window (dk = 1) with k ranging from 2 to 14.5 Å^–1^ and R from 1 to 3.5 Å.

To account for the water effect on XAFS spectra at near-neutral to alkaline pH, the references used for LCF as end-members were the samples of initial magnetite aged in water for 7 days at pH 8.2 and those of maghemite aged in water for 7 days at pH 8.4.

## 3 Results and Discussion

### 3.1 Magnetite and Maghemite Characterization

TEM images of the initial as-synthesized magnetite and maghemite references indicated that both types of NPs consisted of single-crystal domain particles with an average diameter of 10 ± 1 nm and 10 ± 3 nm, respectively (Figure 1).

**Figure 1:**
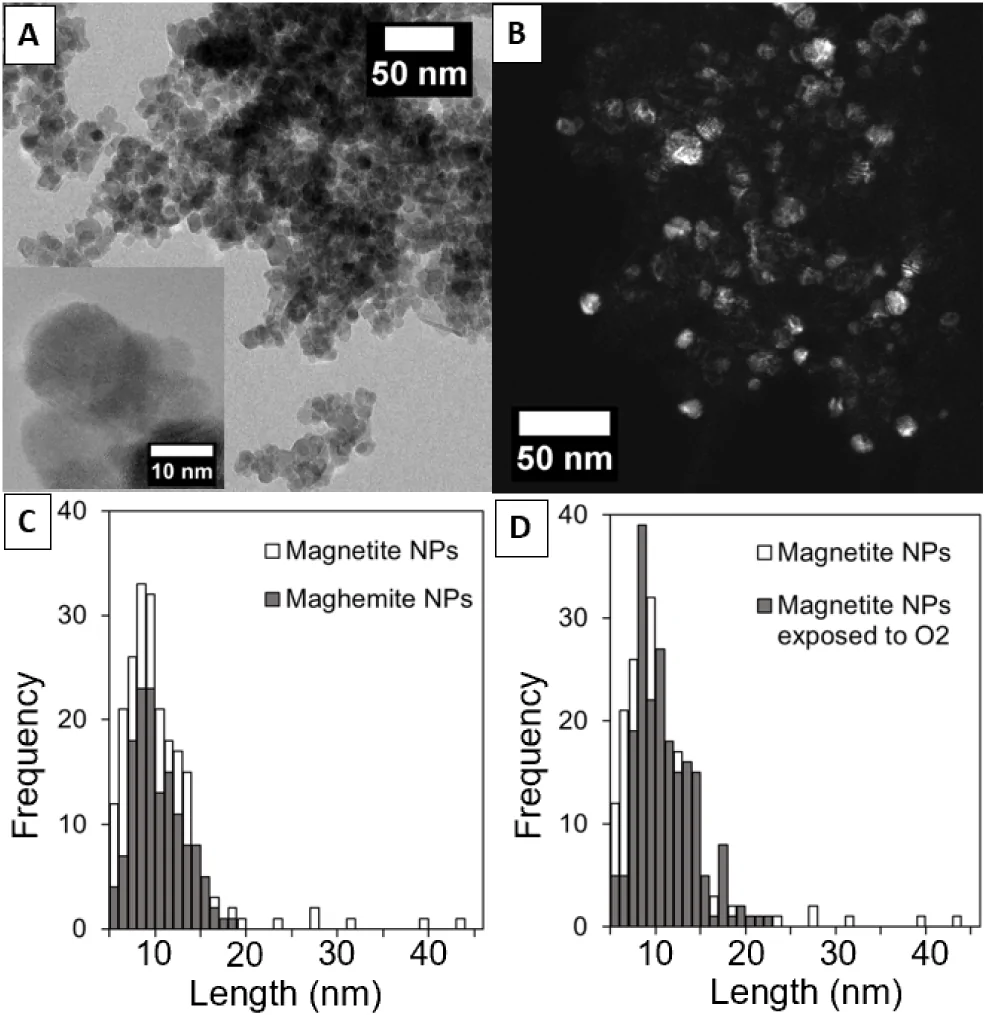
TEM micrographs of the original unreacted as-synthesized magnetite NPs in bright field mode (A) and in dark field mode (B). (C) Size frequency distribution of particle sizes derived from TEM images of synthesized reference maghemite NPs (dark gray, *n* = 139), compared to original unreacted as-synthesized magnetite NPs (in white, *n* = 215). (D) Size frequency distribution of particle sizes of magnetite NPs exposed to 230·10^–6^ M O_2_ (dark gray, *n* = 201), compared to original unreacted as-synthesized magnetite NPs (in white, *n* = 215).

The oxidation state of Fe in the as-synthesized magnetite reference was estimated at 2.74 ± 0.05 using the XANES E_0_, corresponding to R of approximately 0.35 (Figure 2, ESI-Table 1, ESI-Figure 1, ESI Discussion Section 1). The theoretical oxidation state of pure magnetite particles is 2.67 (or R = 0.5). As reported by Jungcharoen et al. (2022),^12^ preferential Fe^2+^ loss during washing has been shown to decrease magnetite stoichiometry, and other studies minimized this effect by performing the washing step under alkaline conditions. In the present study, the washing step was not performed under controlled pH, which accounts for the higher oxidation state observed in our magnetite NPs. Furthermore, this effect is more pronounced when working with NPs, particularly smaller ones, due to their increased surface area-to-volume ratio compared to larger particles.^62^ It is thus important to note that the as-synthesized magnetite NPs used in this study are not stoichiometric magnetite. Based on the XANES-derived oxidation state (2.74 ± 0.05), the Fe^2+^/Fe^3+^ ratio was estimated to be approximately R ≈ 0.35, lower than the theoretical value of R = 0.5 for ideal stoichiometric magnetite. Consequently, all oxidation extents reported in this study should be interpreted relative to the experimentally characterized as-synthesized NPs rather than relative to ideal stoichiometric magnetite.

**Figure 2:**
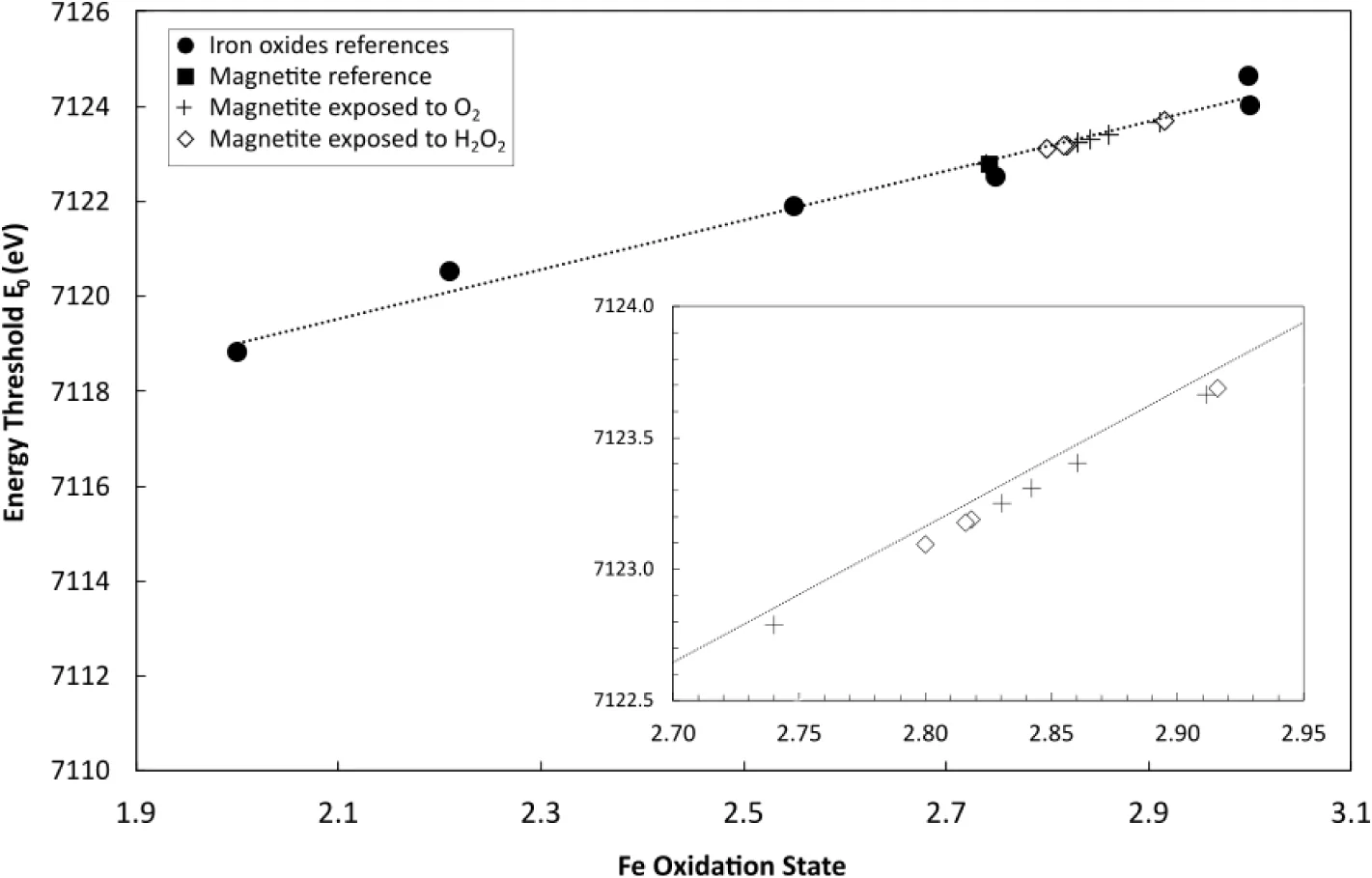
Energy threshold E_0_ as a function of the average Fe oxidation state of references (black circles) and magnetite exposed to various concentrations of O_2_ (crosses) and H_2_O_2_ (diamonds).

Comparison with a stoichiometric micrometer magnetite reference showed measurable differences in the XANES spectra. These differences indicate that the particle size, structural disorder, surface effects, and stoichiometry all contribute to the spectral response. Therefore, the use of a bulk stoichiometric magnetite reference as a quantitative end member would introduce additional size-dependent effects into the calibration. For this reason, the experimentally characterized as-synthesized nanoparticles were retained as the initial reference materials throughout this study. Therefore, the oxidation degrees reported here describe the relative transformation of this experimentally characterized starting material toward maghemite and should not be interpreted as oxidation relative to that of ideal stoichiometric magnetite.

Further characterization of the XANES and EXAFS patterns enabled us to evidence structural differences between initial as-synthesized magnetite NPs and reference maghemite. These characteristics will further be exploited to evaluate the degree of transformation for magnetite particles that were exposed to various oxidizing conditions: on one hand, XANES spectra of initial as-synthesized magnetite and maghemite references showed distinct features demonstrating that XANES LCF can be used to differentiate the oxidation extent of the initial as-synthesized magnetite (ESI-Discussion Section 1, ESI-Figures 2 and 3, ESI-Table 1 and 2). On the other hand, EXAFS showed a decrease in the interatomic distances and the average coordination number (CN) in the Fe_tet_–Fe_oct_ pairs when comparing initial as-synthesized magnetite to maghemite references. This is related to the formation of disordered vacancies in the maghemite structure during oxidative transformation (ESI-Discussion Section 1).

We confirmed XAFS interpretations through the analysis of XRD patterns of initial as-synthesized magnetite and maghemite reference materials. Although the two initial phases have almost identical spinel crystal structures and XRD patterns (Figure 3A), Rietveld analysis of the initial as-synthesized magnetite revealed a cell parameter of 8.3948 Å and a crystallite size of 12 nm, while maghemite was refined and gave a unit cell length of 8.3410 Å and a crystallite size of 10 nm. As a consequence of the decrease in unit cell length, maghemite reflections are shifted to a higher angle compared to initial magnetite. The positions of the reflections (311), (400), (511), and (440) are at 2.480 Å^–1^, 2.989 Å^–1^, 3.888 Å^–1^, and 4.234 Å^–1^ for initial magnetite and 2.494 Å^–1^, 3.008 Å^–1^, 3.915 Å^–1^, and 4.263 Å^–1^ for maghemite, respectively. The maximum peak intensity positions, therefore, allow a clear discrimination between the initial as-synthesized magnetite and the fully oxidized maghemite, and the XRD reflections of partially oxidized magnetite particles (e.g., exposed to O_2_ and H_2_O_2_) are located in between the ones of the initial as-synthesized magnetite and maghemite reference (in red in Figure 3B).

**Figure 3:**
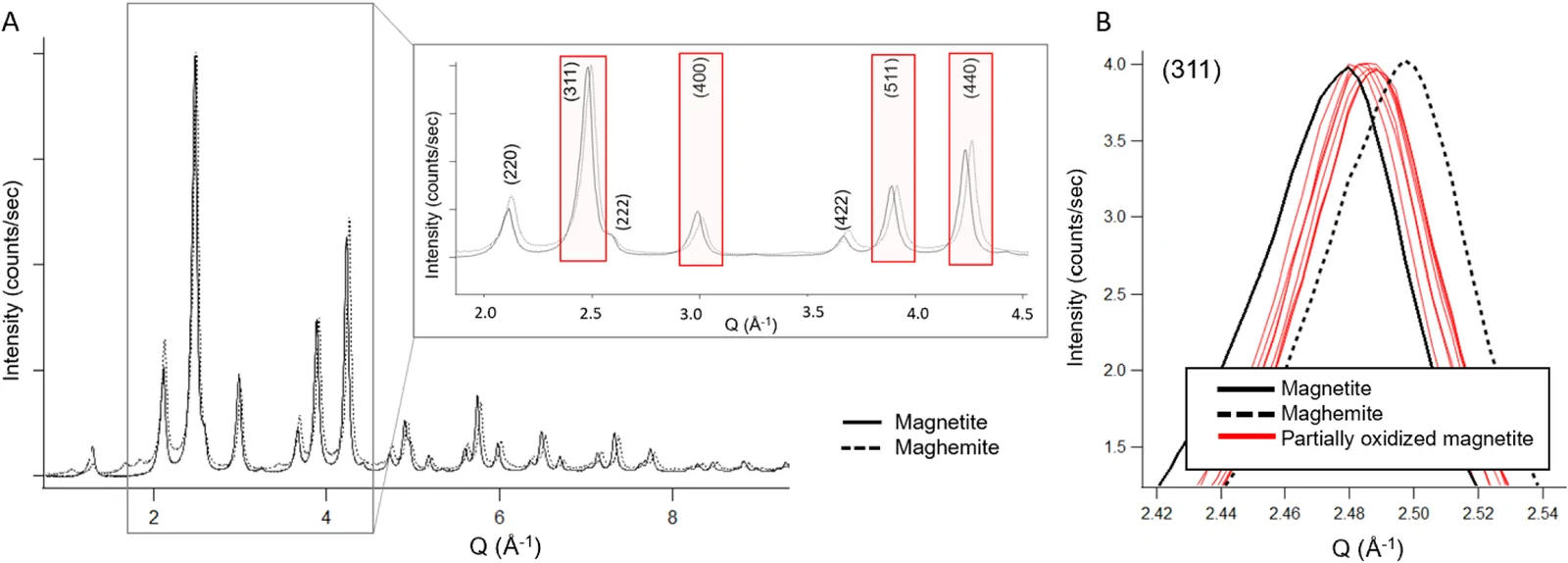
(A) X-ray diffraction patterns of the original unreacted as synthesized magnetite and reference maghemite NPs. (B) Maximum peak intensity positions of reflection (311) of reference magnetite and maghemite and partially oxidized magnetite with dissolved O_2_ and H_2_O_2_.

### 3.2 XRD Data Evaluation and Validation

To utilize XRD routinely to quantify the initial as-synthesized magnetite oxidation extent, we tested and implemented a robust XRD routine. This allowed us to evaluate the magnetite oxidation extent of nanoparticles exposed to O_2_ and H_2_O_2_ and compare the oxidation degree of the different partially oxidized magnetite NPs obtained through XANES. The results for the O_2_ and H_2_O_2_ oxidation extent are presented in the following sections (III.3). XANES LCF (ESI-Figure 2) allowed us to determine the ratio of initial as-synthesized magnetite and maghemite and directly compare it with the degree of transformation obtained from the XRD measurements based on peak shift of four different XRD reflection positions (311, 400, 511, and 440)^11,42,63,64^ and *P*4_3_32 cubic Rietveld refinement model representing maghemite and its partial formation state from initial magnetite. The *P*4_3_32 model was used after evaluating all other available maghemite models (out of the ones listed in II.4), as it was found to be the most appropriate.

Rietveld refinements show that although the lattice parameter is very sensitive to partial oxidation of initial as-synthesized magnetite to maghemite, the Fe site occupancy that should decrease as oxidation progresses shows no clear trend. The exact reasons are unclear, but it is likely a combination of sample properties together with assumptions made during refinement (see ESI Section 2 for full discussion). The vacancy ordering expected with *P*4_3_32 structure was certainly developed very weakly (see ESI Figure S9).

When initial as-synthesized magnetite particles were oxidized either by O_2_ or H_2_O_2_, the resulting modeled composition of the particle mixture became enriched in maghemite (Figures 4 and 5). At all tested conditions, the trends of the transformation extent obtained from XANES LCF were confirmed by XRD analyses, but the value of the evaluated transformation extent varied. Pearson χ^2^ tests were used to assess the goodness-of-fit of the various analysis methods used. The results allowed us to select the best XRD approach to quantify the transformation extent in our samples and to compare them to the LCF results from the XAS analyses (Table 1). The comparison showed that from XRD, the χ^2^ for the oxidation extent ranged from 0.0418 to 0.2240 for peak position at maximum intensity without fitting (Table 1, Figures 4 and 5) and from 0.0673 to 0.5675 when the peaks were modeled with a pseudo-Voigt profile (Table 1, ESI-Figures 5 and 6). Rietveld refinement shows a χ^2^ value of 0.176 or 0.045 if the single biggest outlier is excluded. Exclusion of the biggest outlier was considered justified since the fitting errors of this specific sample, especially the peak position misfits of 551 and 440 peaks (see ESI Discussion Section 2), differed from the other samples; the cause is unknown.

**Figure 4:**
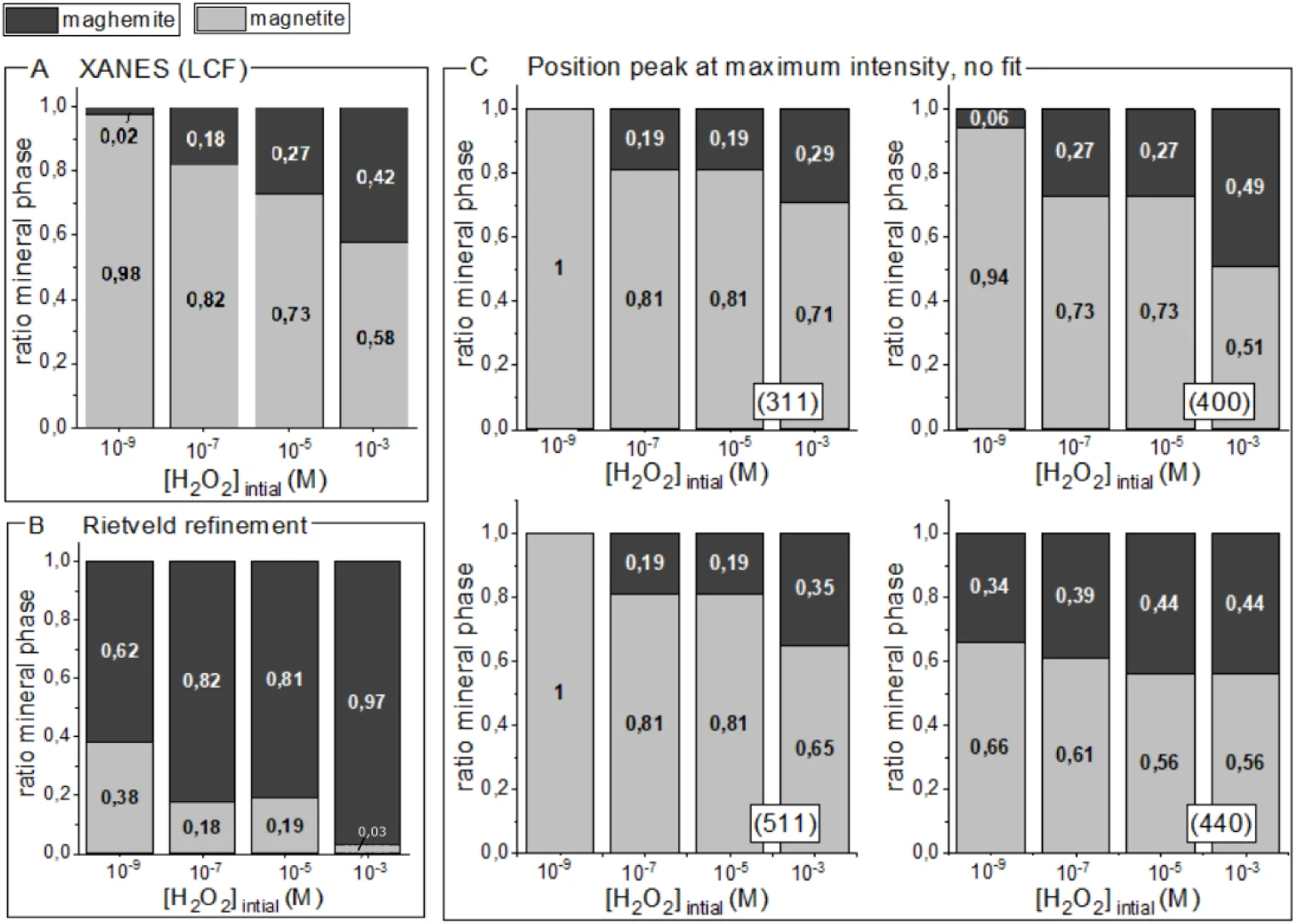
Comparison of the transformation extent (modeled as an initial magnetite-maghemite ratio) of particles exposed to H_2_O_2_. “Magnetite” represents here the initial as-synthesized magnetite NPs. The ratios are obtained by (A) XANES LCF, (B) Rietveld refinement using the P4332 model, and (C) the peak positions of 4 different XRD reflections without fit. Corresponding XANES LCF data and fit are presented in ESI-Figure S9.

**Figure 5:**
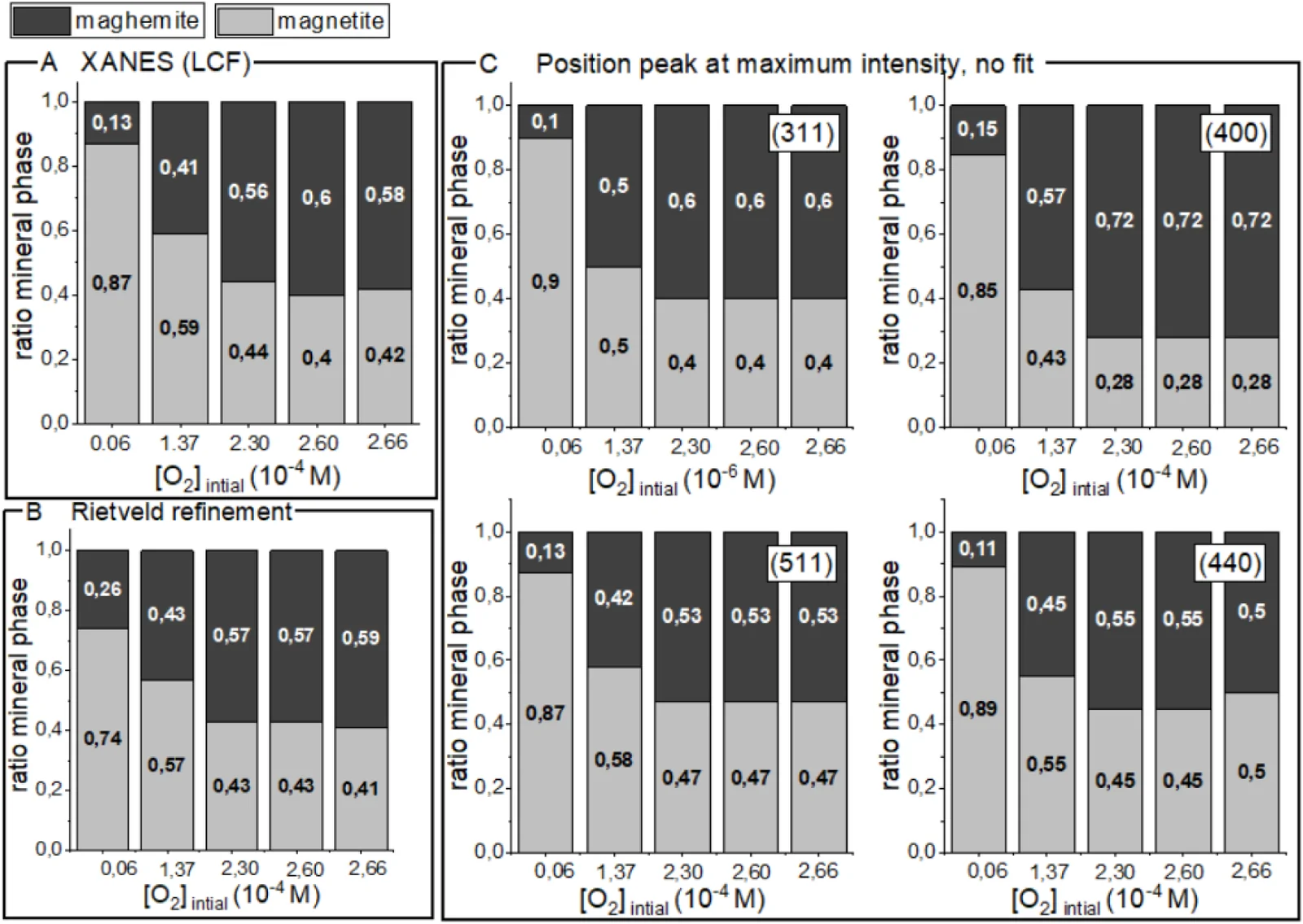
Comparison of the transformation extent (modeled as an initial magnetite-maghemite ratio) of particles exposed to O_2_. “Magnetite” represents here the initial as-synthesized magnetite NPs. The ratios are obtained by (A) XANES LCF, (B) Rietveld refinement using the P4332 model and (C) the peak positions of 4 different XRD reflections without fit. Corresponding XANES LCF data and fit are presented in ESI-Figure S9.

**Table 1:** Summary Results from Goodness-of-Fit χ^2^ Assessment of Transformation Extent Evaluated by XANES LCF and XRD-Derived Data

**XRD analyzing method**		**χ^2^**
position peak at maximum intensity: no fit	_(311)	0.0536
	_(400)	0.1958
	_(511)	0.0418
	_(440)	0.2241
position peak at maximum intensity: pseudo Voigt fit	_(311)	0.0819
	_(400)	0.2090
	_(511)	0.0673
	_(440)	0.5675
Rietveld refinement		0.045

Peak position shifts for some XDR peaks (i.e., the 511 or 311 peaks) have been documented before, but those measurements were never compared to one another or to a different, independent method based on separate parameters such as LCF XANES. Hounslow and Maher (1999)^41^ used the 511 reflection to estimate magnetite unit cell length changes, but they used a method described in Keefer and Shive (1981) developed for transformations of titanomagnetite to titanomaghemite based on the 311 peak analysis, and not the 511 peak.^40^ In addition, Jørgensen et al. (2007)^42^ followed changes in the 311 peak position for maghemites synthesized by thermal decomposition of an amorphous precursor, but they did not provide any independent validation of their results. In the present study, we documented and validated a method that can be applied to quantitatively determine the extent of transformation of magnetite using four different XRD reflection peak positions and a Rietveld refinement method.

According to the lowest χ^2^ value, among all the XRD analysis methods tested, the lattice parameter determination using position shift of the reflection 511 (no fit) was the most effective to estimate the initial as-synthesized magnetite oxidation extent (Table 1). When considering the inherent uncertainty related to XANES LCF method (15%), the single-peak fitting results from 311 and 440 peaks can also be reproduced, within error, from the XANES LCF results (Table 1 and Figure 6).

**Figure 6:**
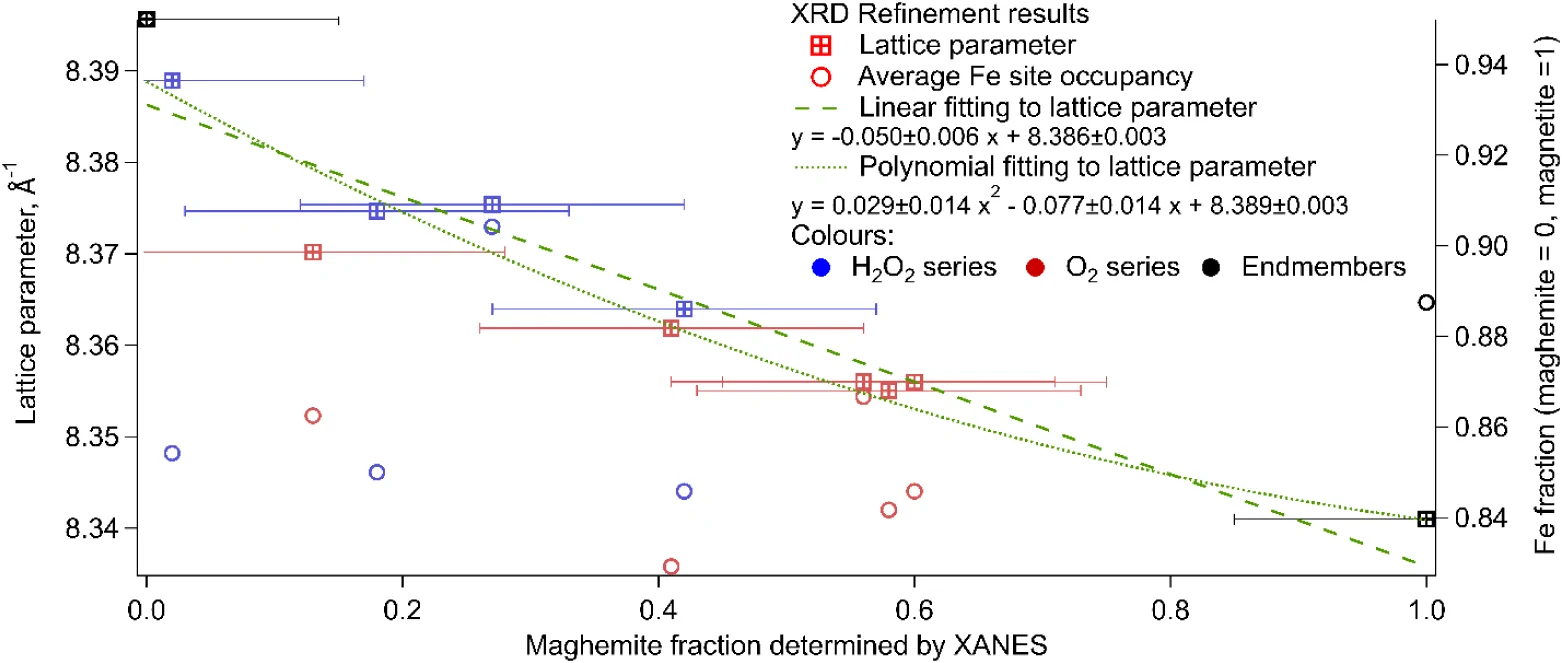
Lattice parameters and Fe occupancy-based degree (0 = initial as synthesized magnetite, 1 = maghemite) as obtained from the Rietveld refinement using *P*4_3_32 model versus oxidation degree measured with XANES (0 = initial as synthesized magnetite, 1 = maghemite). Both, a linear and quadratic, fits show a good fit to the lattice parameter; however, the quadratic fit fits all data points within XANES error of 0.15 maghemite fraction (0.15 value chosen as the minimum certainty in XANES accuracy; see discussion ESI Section 1). Fe fraction does not show a clear trend versus oxidation degree. The samples that were oxidized with H_2_O_2_ are shown in blue, O_2_ in red, and the initial as synthesized magnetite and maghemite endmembers are shown in black.

Similarly, the lattice parameter as determined by Rietveld refinement can also reproduce the maghemite fraction as measured by XANES LCF (Figure 6). However, the Fe site occupancy, also obtained by Rietveld refinement, cannot be used for this purpose. The χ^2^ values of the methods discussed vary over three times; however, all methods are limited by the accuracy with which the maghemite fraction can be determined by XANES. It is assumed by all methods that the XANES result is the correct one to be replicated. If the uncertainty of XANES (≈15%, see ESI discussion Section 1) is considered, all methods, with the exception of the 400 single-peak fit, can reproduce the XANES results, and the χ^2^ values do not carry excessive significance (Figure 7). 511 single-peak fitting is the fastest and easiest to implement and has been used before.^11^ However, Rietveld refinement should be considered the most accurate method to evaluate the oxidation extent of our initial as-synthesized magnetite NPs, as it considers all peaks simultaneously, instead of relying on a single reflection. Any instrument effects, such as those arising from sample displacement, can also be identified and corrected during a refinement, while during a single peak fitting such deviations are likely to be missed. The magnetite-to-maghemite conversion degree in the maghemite fraction could be calculated as f_Mgh_ = 19854.9 – 4726.6a + 281.3a^2^, where a is the lattice parameter in Å. The empirical relationship was obtained by fitting lattice parameters derived from Rietveld refinement against maghemite fractions independently determined by XANES linear combination fitting. The uncertainty associated with the calculated maghemite fraction is estimated to be approximately ±0.15, reflecting primarily the uncertainty of the XANES-LCF calibration data used to establish the relationship rather than the uncertainty of the lattice parameter refinement itself. Problems with sample crystallinity, instrument, sample displacement, and whether it can be described with a simple cubic model would become apparent during a refinement, but not necessarily if a single peak fit is being used. The Rietveld method is not, however, as easily automated as the individual peak fitting-based methods, as false minima are occasionally encountered. Rietveld refinement also seemed to struggle due to the highly complex nature of the sample, which did not seem to be perfectly described by a cubic model. Analysis of peak misfits showed that generally the more oxidized samples produced more errors when refined with a single cubic *P*4_3_32 phase, indicating their more complex nature (see ESI discussion Section 2 for more details).

**Figure 7:**
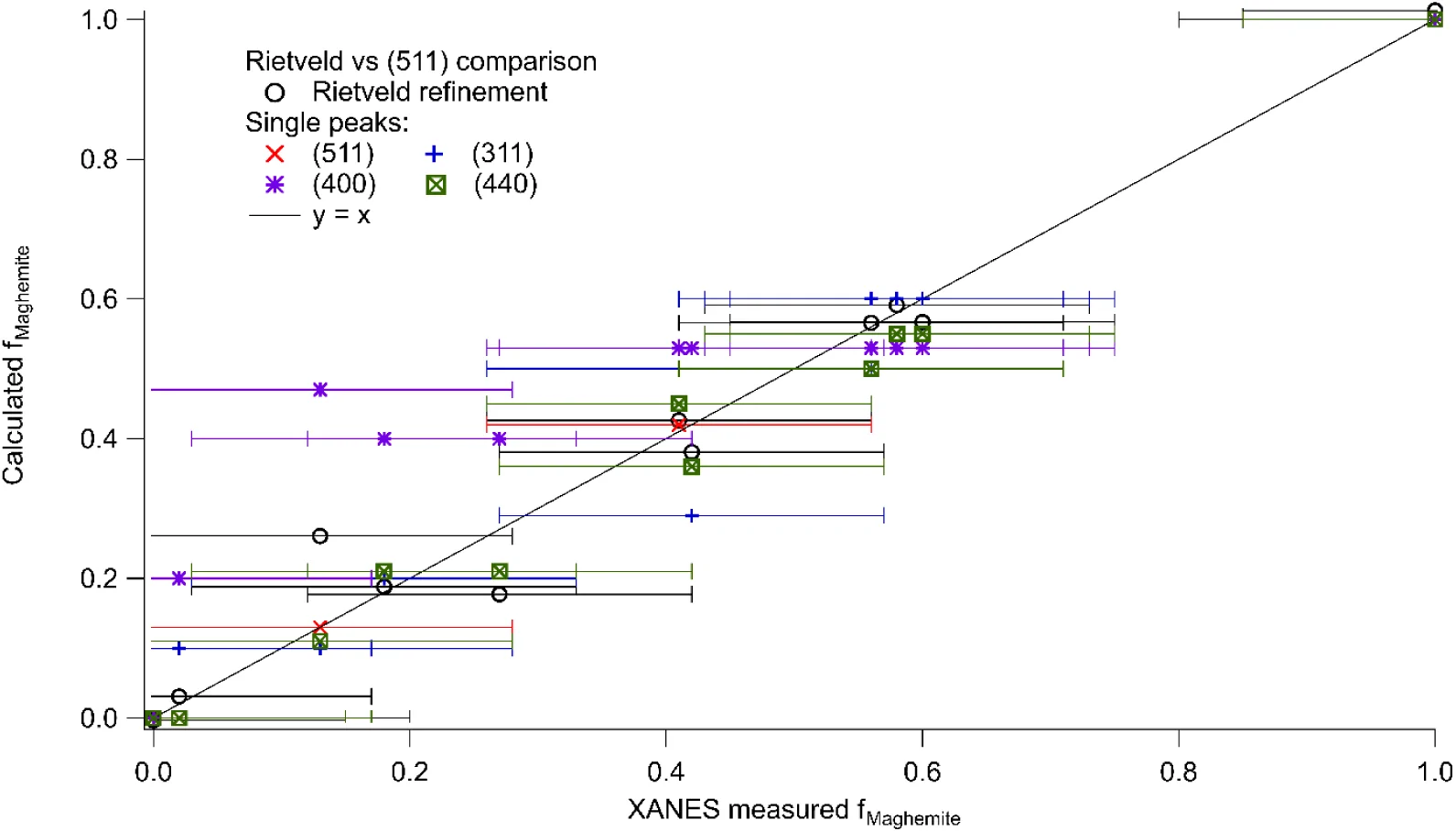
Maghemite fraction calculated from Rietveld refinement and single XRD peak fitting. Error bars shown are 0.15 in the XANES-determined magnetite fraction. Out of the methods tested, the lattice parameter from Rietveld refinement and individual fits to 511, 311, and 440 peaks can, within error, reproduce the maghemite fraction measured by XANES.

### 3.3 Role of Aqueous Oxidants

Analyses of magnetite NPs exposed to various oxidizing conditions showed comparable size range distributions to the initial as-synthesized magnetite and maghemite NPs, as observed previously.^12^ Magnetite NPs exposed to 2.30·10^–4^ M O_2_ had a diameter of 10 ± 1 nm (Figure 1D), equivalent to the initial as-synthesized magnetite NP diameters. Although the size range is similar among the initial NPs and the NPs reacted at various oxidizing conditions, the varying XANES E_0_ (Figure 2) revealed that the magnetite NPs also oxidized to different degrees. The Fe oxidation state of the magnetite NPs exposed to dissolved O_2_ and H_2_O_2_ ranged between 2.7 and 3, corroborated by the calibration curve fitted to iron oxide reference materials (R^2^ = 0.98, ESI-Figure 1, ESI-Table 1). The magnetite-to-maghemite ratios discussed herein are based on the maximum peak position shift of the reflection (511) (no fit), as determined by the XRD approach closer to XAFS results.

#### 3.3.1 Influence of O_2_

The 2 to 66% decrease in aqueous O_2_ after 7 days of exposure suggests that magnetite NPs scavenged the dissolved O_2_ from the solution.

The percentage of maghemite derived from XRD (511) peak (Figure 5C) increased from 13% for the lowest O_2_ concentration to 53% at the higher dissolved oxygen concentrations. Our data seem to indicate that for initial concentrations higher than 2.30·10^–4^ M, the degree of initial as-synthesized magnetite transformation reaches an apparent plateau over the investigated time scale (maximum transformation of 53%). Using the relation between E_0_ and the oxidation state, we estimated the average Fe oxidation states of 2.74 ± 0.05, 2.86 ± 0.05, 2.83 ± 0.05, and 2.84 ± 0.05 and a maximum of 2.91 ± 0.05 for solutions containing initially 0.06, 1.37, 2.30, 2.60, and 2.66·10^–4^ M of dissolved oxygen, respectively (ESI-Table 1).

Complementing the decrease in dissolved oxygen in solution, the results of LSF of EXAFS spectra indicate a concomitant decrease in both Fe–O interatomic distance and Fe_tet_–Fe_oct_ bonding (average CN and bond length) compared to the initial as-synthesized magnetite (see ESI-Table 2 and ESI-Figure 7B and C for data and associated fit). However, the value estimated through LSF does not reach the value calculated for pure maghemite, indicating only a partial structural transformation.

Our results demonstrate that dissolved O_2_ drives the oxidation of magnetite nanoparticles through direct Fe^2+^ → Fe^3+^ conversion, as evidenced by the decrease in aqueous O_2_ (up to 66% after 7 days), the increase in maghemite fraction from 13% to 53% with rising O_2_ concentration, and the corresponding rise in average Fe oxidation state from 2.74 to a maximum of 2.91. However, the transformation reaches a plateau at ∼53% maghemite for [O_2_] > 2.30 × 10^^–^4^ M, suggesting incomplete oxidation, as supported by EXAFS analysis. The apparent plateau observed here over the experimental time scale should not be interpreted as evidence of final oxidation equilibrium. Indeed, long-term studies have shown that magnetite oxidation may continue over longer periods through slower Fe^2+^ redistribution and diffusion-controlled processes.^65^ The reduced Fe–O distances and Fe_tet_–Fe_oct_ bonding, which do not achieve values typical of pure maghemite, are consistent with a core–shell structure where a maghemite shell passivates the magnetite core.

These observations, obtained through a new approach using XRD, align with trends reported in the literature.^12,43,66^ These studies highlight that the oxidation of magnetite is strongly surface-controlled and often limited by kinetic barriers and passivation layers. Similar to the plateau that we observed, they report incomplete transformation under oxidizing conditions, emphasizing that oxygen availability alone does not guarantee full conversion. Instead, factors such as the particle size, surface reactivity, and structural constraints govern the extent of oxidation. Our findings reinforce these previous observations: even at high dissolved [O_2_], oxidation stalls once a protective maghemite shell forms, corroborating the notion of diffusion-limited transformation described previously.

#### 3.3.2 Influence of H_2_O_2_

The percentage of maghemite increases from 0 to 35% for the XRD (511) peak when the NPs were exposed to solutions containing progressively higher hydrogen peroxide concentrations (between 10^–9^ and 10^–3^ M, Figure 4A, ESI-Figure S8A). This same trend was also confirmed by the calculation of the change in Fe oxidation state, which increased from 2.80 ± 0.05 to 2.92 ± 0.05 with increasing hydrogen peroxide concentration (ESI-Table 1). Finally, the LSF of the EXAFS spectra further confirmed a decrease in both Fe–O interatomic distance and Fe_tet_–Fe_oct_ bonding compared to the initial magnetite values (see ESI-Table 2 and ESI-Figure S9B and C for plotted data and associated fit). Similar to the results from the aqueous O_2_ experiments, our data confirm only a partial oxidation of initial as-synthesized magnetite (max 35%) even at high H_2_O_2_ concentrations.

Previous studies^11,13^ have demonstrated the capacity of H_2_O_2_ to oxidize magnetite NPs to maghemite. Jungcharoen and collaborators (2021)^13^ used XMCD and XAS to directly probe the magnetic sublattices and valence distribution in ∼10 nm magnetite. They reported that H_2_O_2_ can drive the Fe^2+^/Fe^3+^ ratio R down from 0.50 to 0.10. The decrease in R was higher than the one observed here (our inferred R is around 0.18 for an oxidation state of 2.92 at the highest H_2_O_2_ concentration). However, the authors do not indicate the concentration of H_2_O_2_ used, and thus, the lower oxidation extent observed here could be due to the lower concentrations of H_2_O_2_. Indeed, Gorski and Scherer (2010)^11^ also observed magnetite oxidation to maghemite but for higher H_2_O_2_ concentrations than here. In addition, the techniques used were different. The XMCD sensitivity to spin canting and A/B-site occupancy reveals surface recrystallization and electronic redistribution that may precede or exceed the bulk-phase conversion captured by XRD, helping explain why valence changes (low R) can look more pronounced even when complete crystallographic conversion (to pure maghemite) is not achieved. Both studies^11,13^ emphasize that H_2_O_2_ is a strong oxidant and rapidly oxidizes Fe^2+^ at the NPs surface, yet full conversion is typically curtailed by passivating shells and diffusion limits, even under aggressive conditions. This mechanistic framing aligns with the plateaued phase transformation and EXAFS evidence for a core–shell architecture and with Jungcharoen’s observation that H_2_O_2_ drives large decreases in R without necessarily producing complete structural maghemite throughout the particle. Overall, our results, obtained through a new approach using XRD, and these studies concur that shell formation imposes a kinetic ceiling on H_2_O_2_-driven magnetite oxidation.

## 4 Conclusions

Changes in the (311), (400), (511), and (440) Bragg reflections in the XRD patterns, as well as lattice parameter and Fe occupancy determined by Rietveld refinement, have been tested for the determination of the degree of magnetite NP oxidation in aqueous conditions. We showed that 511, 311, and 440 XRD peak shifts and Rietveld refinement-determined lattice parameters can reproduce within the margin of error maghemite fractions measured with XANES within error. The (400) XRD peak shift and Fe occupancy or vacancy concentrations from Rietveld refinement are unreliable among the methods tested. To obtain the oxidation degree (maghemite fraction) from the lattice parameter equation f_Mgh_ = 19854.9 – 4726.6a + 281.3a^2^, where a is the lattice parameter in Å, could be used. The empirical lattice parameter calibration proposed here was established for ∼10 nm partially oxidized magnetite nanoparticles and should not yet be considered universally applicable to magnetite particles of different sizes, morphologies, or initial stoichiometries without additional validation by experimentation. This study highlights how this approach can provide fundamental new insights into magnetite NPs partial oxidation. By using a combination of XAFS and Rietveld analyses of XRD patterns, we showed that the partial oxidative transformation of magnetite proceeds via a distortion of its crystal structure, but we were not able to observe or clearly identify whether separate areas of the NPs were distinctively magnetite or maghemite. Finally, we documented the partial transformation of magnetite NPs to maghemite when exposed to various oxidants. We showed that O_2_ induces an apparent maghemite fraction of approximately 55–60% relative to the as-synthesized magnetite NPS used as the initial reference state.

## Supplementary Material



## Notes

XANES spectra of references (Figure S1); measured E_0_ and corresponding Fe oxidation state (Table S1); XAS characterization of the initial magnetite and maghemite (Discussion section 1); EXAFS LSF parameters (Table S2); XANES and EXAFS plots of initial magnetite and maghemite (Figure S2); plot of LSF results for initial magnetite and maghemite (Figure S3); initial magnetite and maghemite XANES spectra in ultrapure water (Figure S4); XRD fitting results (Figures S5 and S6); plots of XANES and EXAFS LCF and LSF results for O_2_ exposure (Figure S7); plots of XANES and EXAFS LCF and LSF results for H_2_O_2_ exposure (Figure S8); Rietveld refinement (Discussion section 2); superstructure peaks XRD (Figure S9); peak position XRD (Figure S10); comparison of peak profile (XRD) (Figure S11); Rietveld refinements (Figure S12) (PDF)

## #

Department of Chemistry and Biochemistry, Université de Moncton, Moncton, NB, Canada E1A 3E9
