# Supplementary material for: Quantification of Partial Oxidation of Magnetite to Maghemite in Aqueous Solution Using XRD
Source: ACS Omega. 2026 Oct 15;11(42):64404–15. doi: 10.1021/acsomega.6c01180 (PMC13717529; doi:10.1021/acsomega.6c01180)
Supplement: Supplementary file 1 [file ao6c01180_si_001.pdf]

Quantification of partial oxidation of magnetite to maghemite in aqueous solution using XRD

ELECTRONIC SUPPLEMENTARY INFORMATION (ESI)

MORGANE DESMAU [a,†,*,‡], *THAÏS COUASNON* [b,‡], *ROBERTS BLUKIS* [b,c], *JEFFREY PAULO H. PEREZ* [b], *LIANE G. BENNING* [b,d,*]

a. Deutsches Elektronen-Synchrotron DESY, Notkestr. 85, 22607 Hamburg, Germany

b. GFZ, Helmholtz Center for Geosciences, Telegrafenberg, 14473 Potsdam, Germany

c. Leibniz-Institut für Kristallzüchtung, Max-Born Str. 2, 12489 Berlin, Germany

d. Department of Earth Sciences, Freie Universität Berlin, 12249 Berlin Germany

Corresponding Author *morgane.desmau@umoncton.ca, and benning@gfz.de

Present Addresses

† Department of Chemistry and Biochemistry, Université de Moncton, Moncton, NB, Canada E1A 3E9

‡These authors contributed equally. (match statement to author names with a symbol)

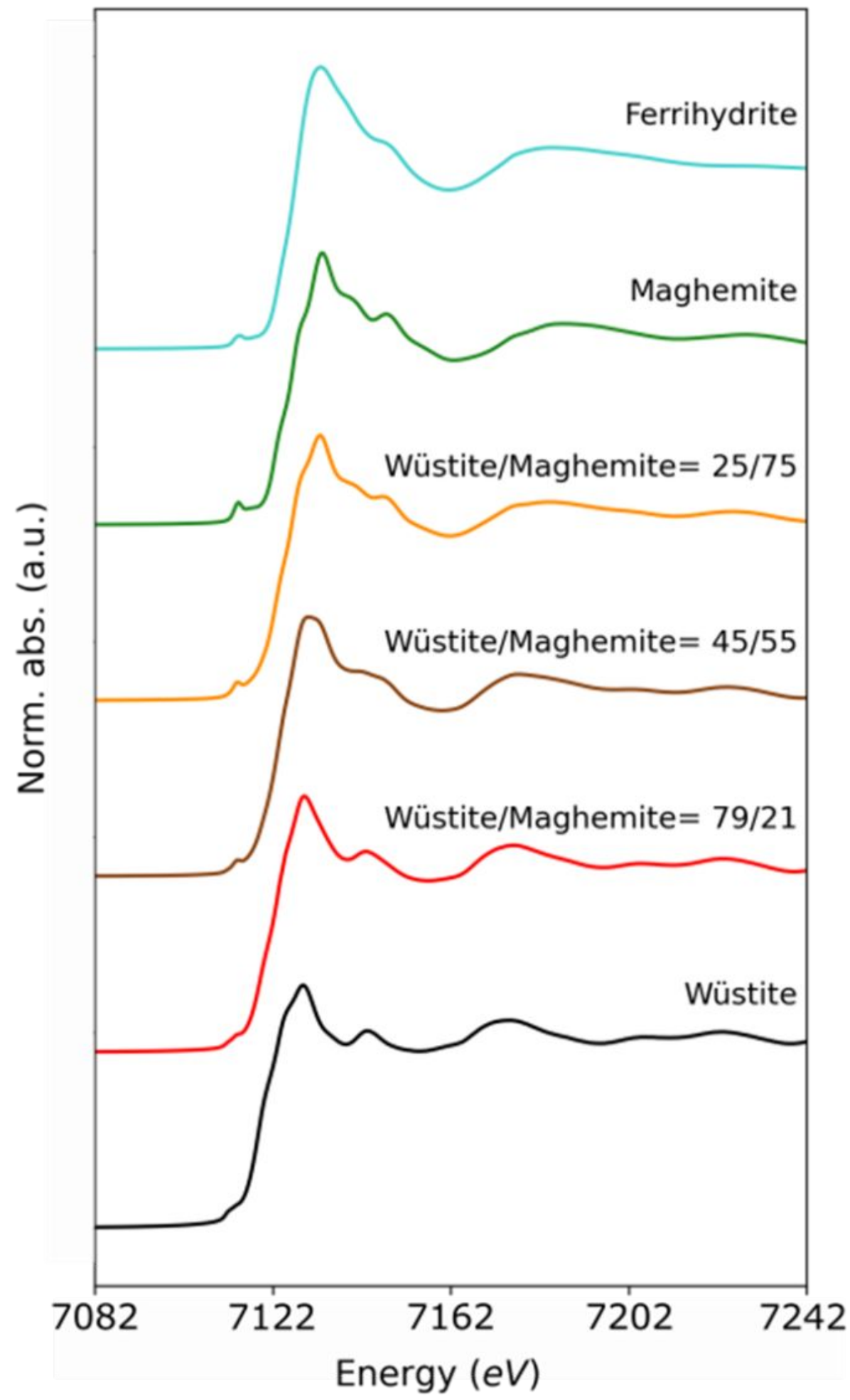

Figure S1: Fe K-edge XANES spectra of iron-based mineral references used to generate a calibration curve to determine the average Fe oxidation state. Some of the references consisted of a mixture of wüstite and maghemite mineral powder at distinct ratios (25/75, 45/55 and 79/21).

Table S1: Measured energy thresholds, E_0, and calculated corresponding Fe oxidation states using the calibration curve shown in Figure 3 in the main manuscript.

	E_0, measured	Oxidation State estimated	Oxidation State calculated
Wüstite	7118.8	2.00	
Wüstite79/Maghemite21	7120.5	2.21	
Wüstite45/Maghemite55	7121.9	2.55	
Wüstite25/Maghemite75	7122.5	2.75	
Maghemite	7124.0	3.00	
Ferrihydrite	7124.6	3.00	
Magnetite	7122.8		2.74
Magnetite, pH = 6.7	7124.1		2.99
Magnetite, pH = 7.2	7123.0		2.78
Magnetite, pH = 8	7122.9		2.76
Magnetite, pH = 8.2	7122.8		2.74
$[O_2]_{initial} = 0.06 \cdot 10^{-4}$M	7122.8		2.74
$[O_2]_{initial} = 1.37 \cdot 10^{-4}$M	7123.4		2.86
$[O_2]_{initial} = 2.30 \cdot 10^{-4}$M	7123.7		2.91
$[O_2]_{initial} = 2.60 \cdot 10^{-4}$M	7123.3		2.84
$[O_2]_{initial} = 2.66 \cdot 10^{-4}$M	7123.3		2.83
$[H_2O_2]_{initial} = 10^{-3}$ M	7123.7		2.92
$[H_2O_2]_{initial} = 10^{-5}$ M	7123.2		2.82

$[H_2O_2]_{initial} = 10^{-7}$ M	7123.2	2.82
$[H_2O_2]_{initial} = 10^{-9}$ M	7123.1	2.80

ESI Discussion section 1: XAS characterization of the initial as synthesized magnetite and maghemite references

XANES characterization of the magnetite and maghemite references

In XANES spectra, the difference in iron oxidation state is visible as the shift to higher energy of spectra as the degree of oxidation increases. To distinguish between the oxidation states of iron in the initial as synthesized magnetite and maghemite references, the energy difference (ΔE) between the spectral features of the two distinct NPs must be resolvable considering the energy resolution of the monochromator at the Fe K-edge (~ 0.7 eV). As explained in the experimental section, E_0 was used as an indicator of the oxidation state and was determined using the half-maximum position in the XANES spectra (ESI-Figure 2B). For magnetite, the average E_0 value is 7122.8±0.1 eV while for maghemite this value increases up to 7124.0±0.1 eV. The ΔE between the two species is 1.2±0.2 eV and is therefore higher than the energy resolution of the monochromator. Considering a linear response in the half-maximum position in the XANES spectra, the oxidation state of Fe should be resolved with an uncertainty of ~20% (Figure 2 in the main text). We therefore estimated the oxidation of Fe in the reference magnetite at 2.74±0.05 (ESI-Table 1). This value is in agreement with previous study of magnetite NPs of 10 nm, or smaller[1]. The difference between the theoretical oxidation state of Fe in magnetite (2.66) and the measured one is due to the higher presence of Fe^{3+} at the magnetite surface when considering NPs instead of micrometric particles[1].

In addition to the difference in E_0 indicating changes in the oxidation state, specific features in the derivatives of the XANES spectra (FD-XANES, in red in ESI-Figure 2 B, highlighted by arrows) of maghemite reveal, after a first peak around 7113.3 eV, corresponding to the Fe(1s→3d/4p) electron transition pre-edge peak, a

small shoulder at 7116.5 eV. Then, three variable intensity peaks at 7122.5 eV, 7126.5 eV and 7131.3 eV are visible. These peaks are common features of Fe absorption K-edge spectra and are attributed to different Fe electronic transitions and phenomena of charge transfer between molecular orbitals (Fe-O)[2]. On the contrary, in the XANES and FD-XANES spectra of magnetite (in black ESI-Figure 2 A and B), noticeable are shifts to lower energy and the missing shoulder at 7116.5 eV. In addition, the three peaks are also different, the first peak became a vague shoulder and the two other peaks are of equal intensity. However, the specific patterns of the magnetite and maghemite spectra allowed us to use LCF to analyze the spectra and determine the time resolved change in Fe speciation and to estimate the transformation extent of partially oxidized magnetite NPs modeled as a ratio of magnetite/maghemite.

EXAFS characterization of the magnetite and maghemite references

Although, the EXAFS frequencies are relatively similar for both magnetite and maghemite, specific features in the magnetite spectra are distinct, specifically between 8 and 12 $Å^{-1}$ (ESI-Figure 2 C, D). In our study, the amplitude difference in k and R spaces between the magnetite and maghemite spectra are consistent with the presence of vacancies and a more disordered structure in the later[3].

In the Fourier-transform EXAFS signal $\chi(R)$ in ESI-Figure 2D, the first main contribution, between 1 and 2 Å (uncorrected for phase-shift), corresponds to the first nearest neighbors, Fe-O pair. This contribution has been modeled using Levenberg-Marquadt non-linear least-squares fitting (LSF, ESI-Figure 3, ESI-Table 3). The Fe-O distance corresponding to the Fe-O octahedron and to the Fe-O tetrahedron coordination cannot be separately resolved in this study. However, the average fitted Fe-O distance for magnetite (2.03±0.01 Å) and maghemite (1.94±0.01 Å) are consistent with the average values expected from crystallographic data and from the literature[4,5].

In addition, we calculated a smaller value for the coordination number ($CN_{(Fe\text{-}O)}$) of maghemite (4.85±0.66) compared to initial as synthesized magnetite (6.7±1.2, see ESI-Table 3) using LSF. Given the high uncertainties in $CN_{(Fe\text{-}O)}$ estimates (around 30%) a decrease in $CN_{(Fe\text{-}O)}$ is always difficult to interpret. However, in our case, this decrease is correlated with a decrease in the interatomic distance and Debye-Waller factor,

indicating a likely significant decrease of $CN_{(Fe-O)}$. The $CN_{(Fe-O)}$ for magnetite and maghemite, are however within error of XRD Rietveld refinement values (5.33 and 5.25[4]). Nevertheless, the observed minor differences might be explained by the out-of-phase interference between the two Fe-O shells[5] and/or by the average value of S_0^2 chosen to perform the LSF. The second nearest neighbor Fe-Fe pair contribution (between 2 and 3.5 Å, ESI-Figure 2 C, and ESI-Figure 3) shows a clear distinction in the magnetite Fourier transform signal between the two iron sites. The first peak at 2.99±0.02 Å correspond to the Fe-Fe distances interpreted as edge sharing octahedra (Fe_{oct}-Fe_{oct}). The second peak at 3.48±0.01 Å, corresponds to the Fe-Fe distances of a single-corner sharing conformation between a tetrahedron and its nearest octahedron neighbor (Fe_{tet}-Fe_{oct})[4,6,7].

For magnetite and maghemite, the Fe_{oct}-Fe_{oct} interatomic distances, corresponding to the first peak in $\chi(R)$ (Fig ESI-Figure 3), are similar (2.98±0.02 and 2.99±0.02 Å, respectively) and the $CN_{(Feoct-Feoct)}$ (4.1±1.8 and 3.7±1.6, respectively) are also within error, and close to the expected value of 4 for an inverse spinel structure[8]. In the maghemite $\chi(R)$ (in red in ESI-Figure 3), the difference between the two distinct iron sites is less pronounced and the calculated interatomic distance of the Fe_{tet}-Fe_{oct} is smaller: 3.45±0.01 Å. A decrease in CN of the Fe_{tet}-Fe_{oct} second nearest neighbor shell in maghemite was also observed. The concomitant decrease of the interatomic distances and the average CN in the Fe_{tet}-Fe_{oct} pairs are related to the appearance of disordered vacancies in the maghemite structure during the oxidative transformation. Similarly, to what is happening in the first coordination shell (Fe-O bonding) the decrease in the number of Fe atoms in the 2nd coordination shell around Fe favored the decrease of the interatomic distance between Fe_{tet}-Fe_{oct}. The observations from the EXAFS spectra, confirmed that magnetite has longer Fe-O and Fe_{tet}-Fe_{oct} interatomic distances and higher $CN_{(Fetet-Feoct)}$ than maghemite. The decrease in $CN_{(Fetet-Feoct)}$ value is significant because it is associated with the interatomic distance decrease. The present study of intermediate NPs created through partial oxidation should focus mostly on Fe-O and Fe_{tet}-Fe_{oct} bounding (average coordination number and bond length).

Table S2: EXAFS LSF parameters for the magnetite and maghemite references and the magnetite NPs exposed to different concentrations of O_2 and H_2O_2. S^2_o corresponds to the amplitude reduction factor, N to the path degeneracy (corresponding to the coordination number CN), σ^2 to the Debye-Waller factor, R to the path length and ΔE_0 to the shift in energy threshold. The r-factor is used to estimate the goodness of the fit.

Sample	Bond	R	N / CN	ΔE_0	σ^2	S^2_0	r-factor
Initial Magnetite as-synthesized	Fe-O	2.03±0.01	6.7±1.2	1.5±1.3	0.014±0.003	0.79	0.01
	Fe_{oct}-Fe_{oct}	2.98±0.02	4.1±1.8		0.008±0.003		
	Fe_{tet}-Fe_{oct}	3.48±0.01	11.1±2.0		0.007±0.001		
Maghemite reference	Fe-O	1.94±0.01	4.9±0.7	-3.6±1.5	0.010±0.002	0.79	0.012
	Fe_{oct}-Fe_{oct}	2.99±0.02	3.7±1.6		0.010±0.003		
	Fe_{tet}-Fe_{oct}	3.45±0.01	8.0±1.7		0.009±0.002		
	Fe_{tet}-Fe_{oct}	3.48±0.01	11.0±1.9		0.007±0.001		
Magnetite $[O_2]_{initial}=0.06\cdot10^{-4}$ M	Fe-O	2.02±0.01	6.9±1.2	1.3±1.3	0.014±0.003	0.79	0.01
	Fe_{oct}-Fe_{oct}	2.98±0.02	3.9±1.7		0.008±0.003		
	Fe_{tet}-Fe_{oct}	3.48±0.01	11.6±2.0		0.007±0.001		
Magnetite $[O_2]_{initial}=1.37\cdot10^{-4}$ M	Fe-O	1.98±0.01	6.0±0.9	1.4±1.5	0.012±0.002	0.79	0.009
	Fe_{oct}-Fe_{oct}	3.00±0.02	6.7±2.3		0.012±0.003		
	Fe_{tet}-Fe_{oct}	3.48±0.01	7.9±1.6		0.006±0.001		
Magnetite $[O_2]_{initial}=2.30\cdot10^{-4}$ M	Fe-O	1.96±0.01	5.8±0.8	2.6±1.5	0.012±0.002	0.79	0.01
	Fe_{oct}-Fe_{oct}	2.99±0.02	4.9±1.7		0.010±0.003		
	Fe_{tet}-Fe_{oct}	3.47±0.01	8.1±1.6		0.007±0.001		
	Fe-O	1.97±0.01	5.8±0.8	1.7±1.4	0.012±0.002	0.79	0.009
	Fe_{oct}-Fe_{oct}	3,00±0.02	5.7±1.9		0.011±0.002		

Magnetite $[O_2]_{initial}=2.60\cdot10^{-4}$ M	Fe_{tet}-Fe_{oct}	3.48±0.01	7.7±1.5		0.007±0.001		
Magnetite $[O_2]_{initial}=2.66\cdot10^{-4}$ M	Fe-O	1.97±0.01	5.7±0.8		0.012±0.002		
	Fe_{oct}-Fe_{oct}	2.99±0.02	5.1±1.8	2.3±1.4	0.010±0.010	0.79	0.01
	Fe_{tet}-Fe_{oct}	3.47±0.01	7.9±1.5		0.007±0.001		
Magnetite $[H_2O_2]_{initial}=10^{-9}$ M	Fe-O	2.03±0.01	6.7±1.2		0.014±0.003		
	Fe_{oct}-Fe_{oct}	2.98±0.02	4.3±1.9	1.7±1.3	0.003±0.003	0.79	0.01
	Fe_{tet}-Fe_{oct}	3.48±0.01	11.0±2.0		0.001±0.001		
Magnetite $[H_2O_2]_{initial}=10^{-7}$ M	Fe-O	2.02±0.01	7.0±1.3		0.014±0.003		
	Fe_{oct}-Fe_{oct}	2.98±0.02	3.9±1.7	1.4±1.3	0.008±0.003	0.79	0.01
	Fe_{tet}-Fe_{oct}	3.48±0.01	11.7±2.1		0.007±0.001		
Magnetite $[H_2O_2]_{initial}=10^{-5}$ M	Fe-O	2.02±0.01	7.1±1.3		0.014±0.002		
	Fe_{oct}-Fe_{oct}	2.98±0.02	3.8±1.7	1.3±1.3	0.007±0.003	0.79	0.01
	Fe_{tet}-Fe_{oct}	3.47±0.01	12.1±2.1		0.007±0.007		
Magnetite $[H_2O_2]_{initial}=10^{-3}$ M	Fe-O	1.98±0.01	5.8±0.9		0.013±0.002		
	Fe_{oct}-Fe_{oct}	3.00±0.02	6.3±2.1	1.3±1.5	0.012±0.003	0.79	0.01
	Fe_{tet}-Fe_{oct}	3.48±0.01	7.6±1.5		0.007±0.003		
Maghemite pH=8	Fe-O	1.94±0.01	5.4±0.7		0.010±0.002		
	Fe_{oct}-Fe_{oct}	2.99±0.02	3.8±1.7	3.9±1.5	0.011±0.004	0.79	0.011
	Fe_{tet}-Fe_{oct}	3.45±0.01	8.6±1.8		0.009±0.002		

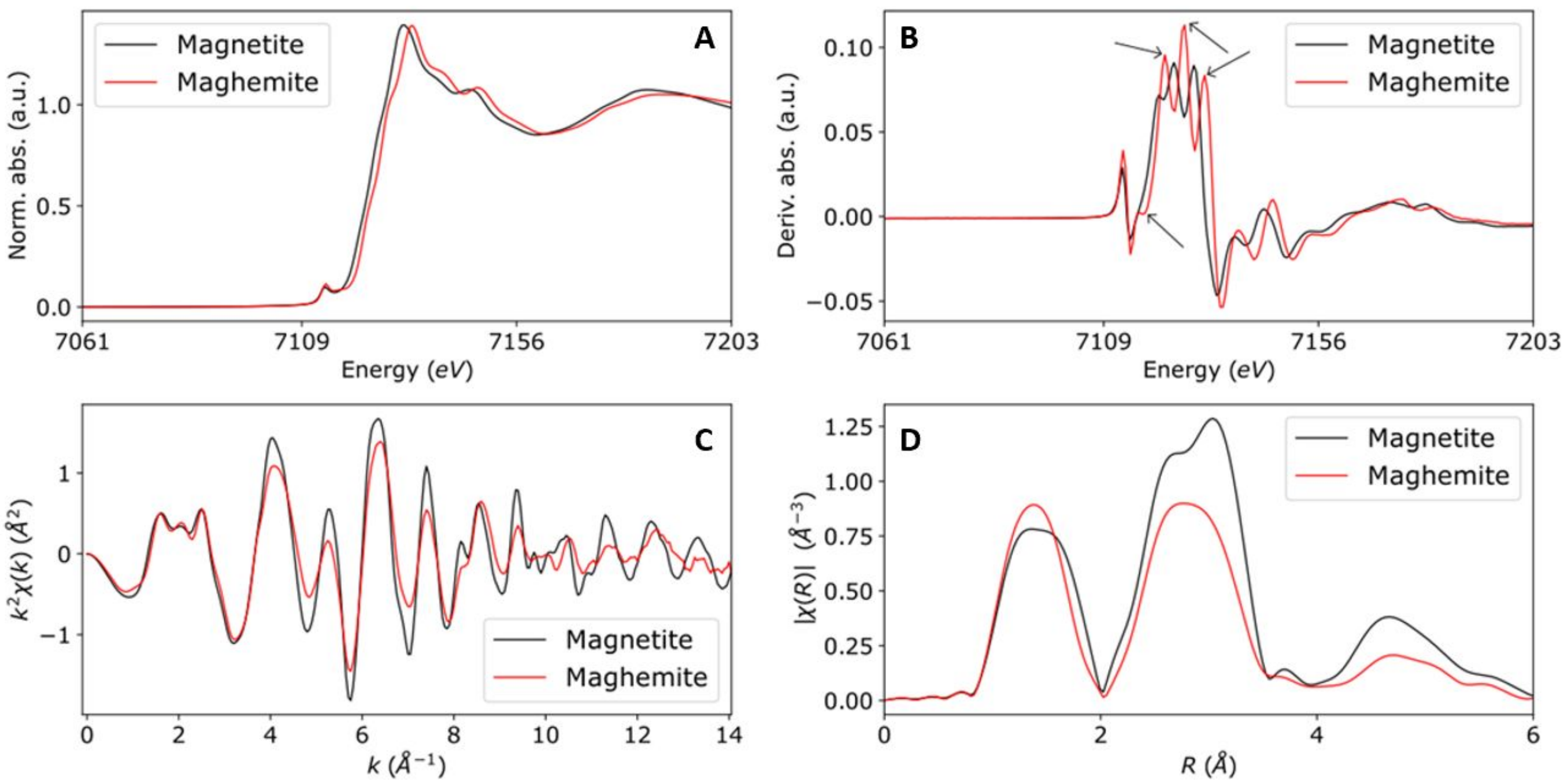

Figure S2: Fe K-edge XANES (A), FD-XANES (B), EXAFS (C) and EXAFS Fourier-transform (D) of initial as synthesized magnetite (black) and maghemite (red) references. In B, the arrows highlight the specific peaks characteristic for maghemite compared to magnetite.

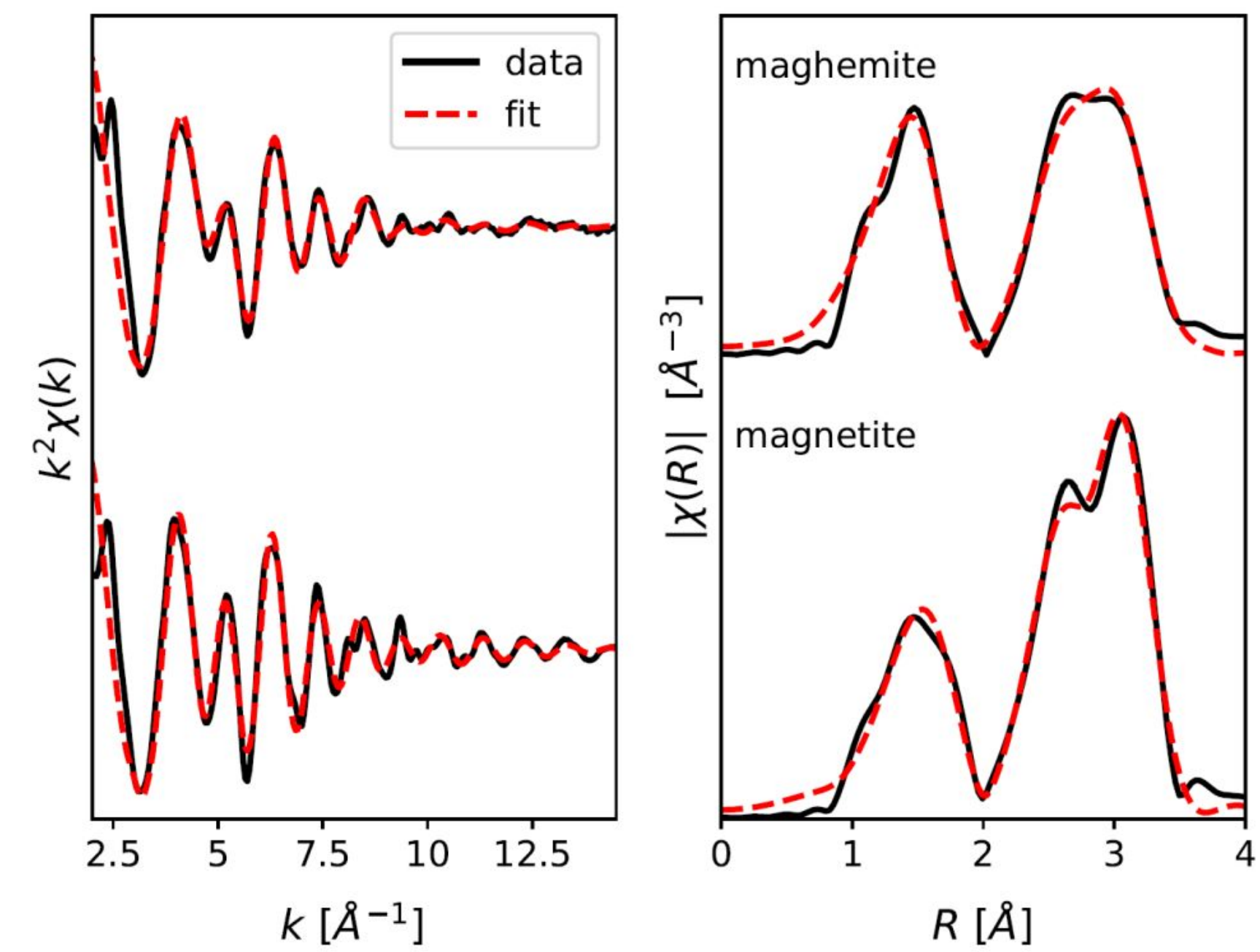

Figure S3: Results of least-squares fitting (LSF) analysis on Fe K-edge EXAFS spectra for initial as synthesized magnetite and maghemite references. EXAFS Fourier-transform is also represented.

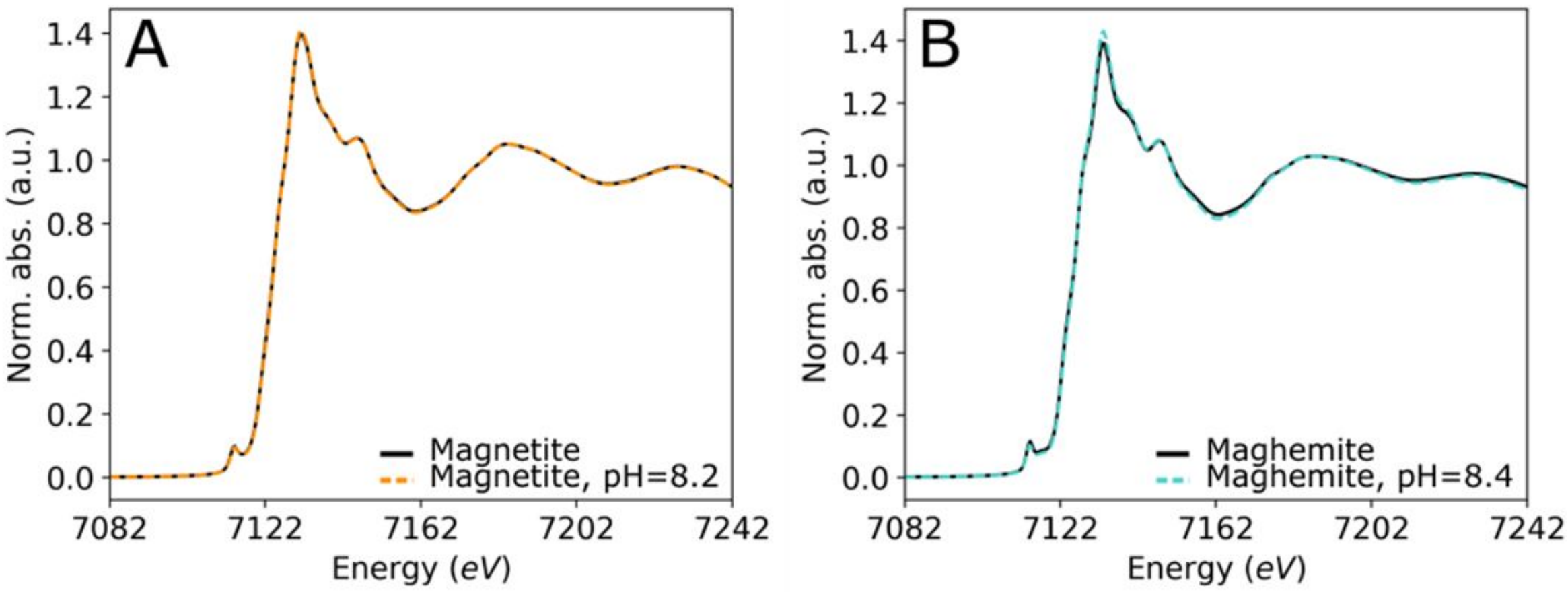

Figure S4: Comparison of (A) Fe K-edge XANES spectra of initial as synthesized magnetite and magnetite exposed to ultrapure water at pH=8.2 and (B) Fe K-edge XANES spectra of maghemite and maghemite exposed to ultrapure water at pH=8.4. No significant differences are visible between the different spectra of magnetite and maghemite. The two XANES patterns are used as reference end members to investigate magnetite exposed in water and to dissolved oxidizing agents.

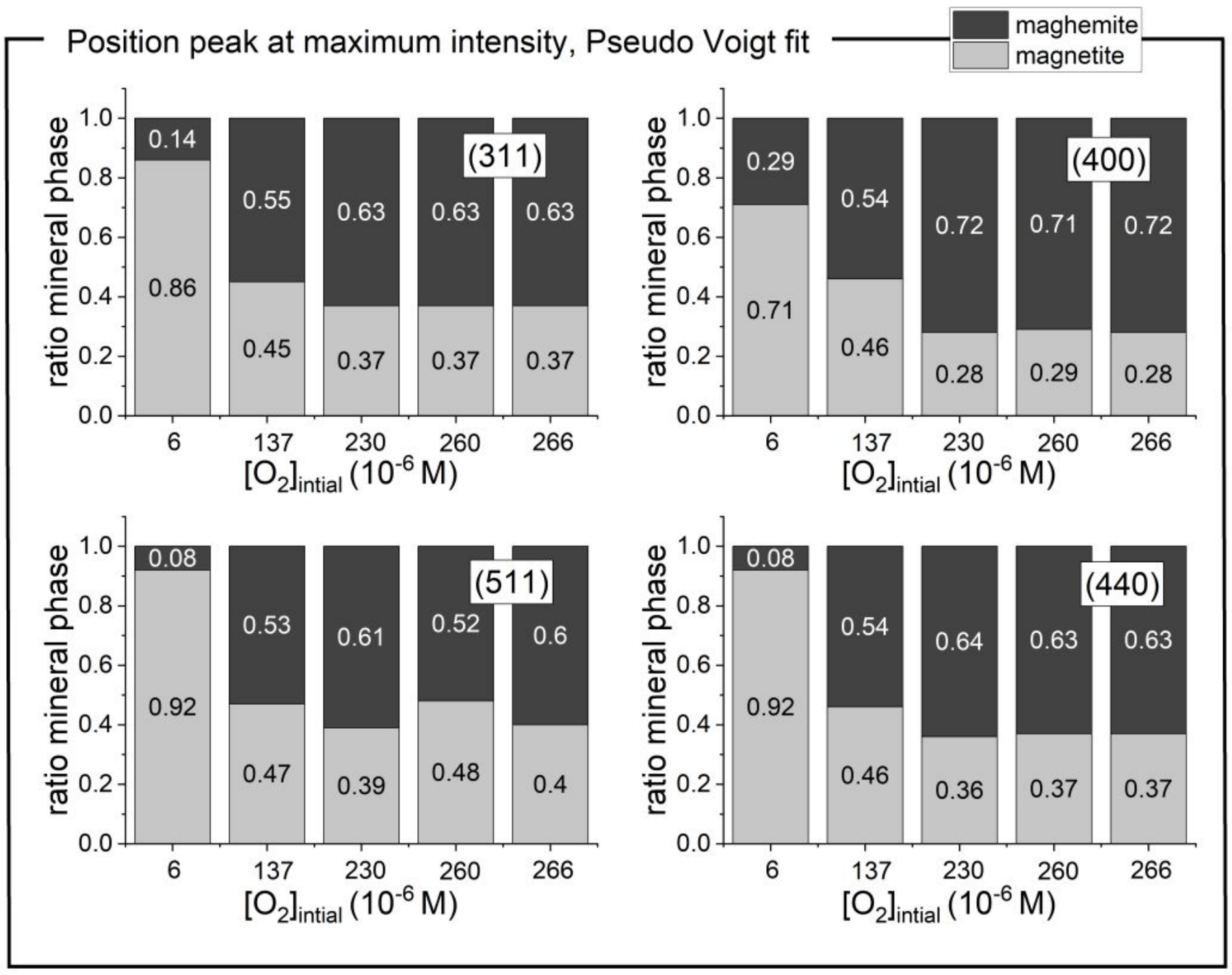

Figure S5: Comparison of the transformation extent (modeled as a magnetite-maghemite ratio) of particles exposed to O_2. The ratio is obtained through the peak position shift evaluated with the pseudo-Voigt fitting of 4 different XRD reflections: (311), (400), (511), (440).

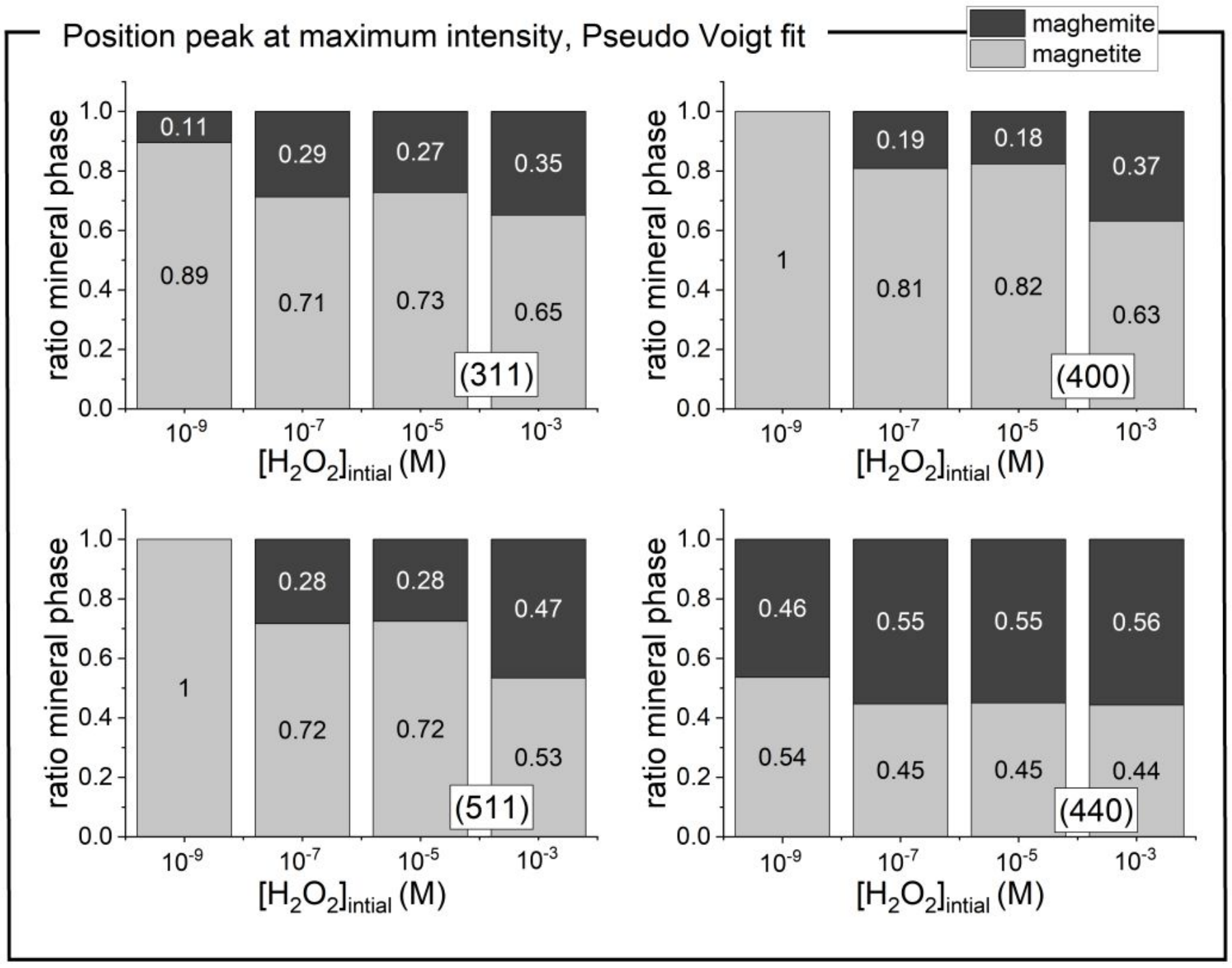

Figure S6: Comparison of the transformation extent (modeled as a magnetite-maghemite ratio) of particles exposed to H_2O_2. The ratio is obtained through the peak position shift evaluated with the pseudo-Voigt fitting of 4 different XRD reflections: (311), (400), (511), (440).

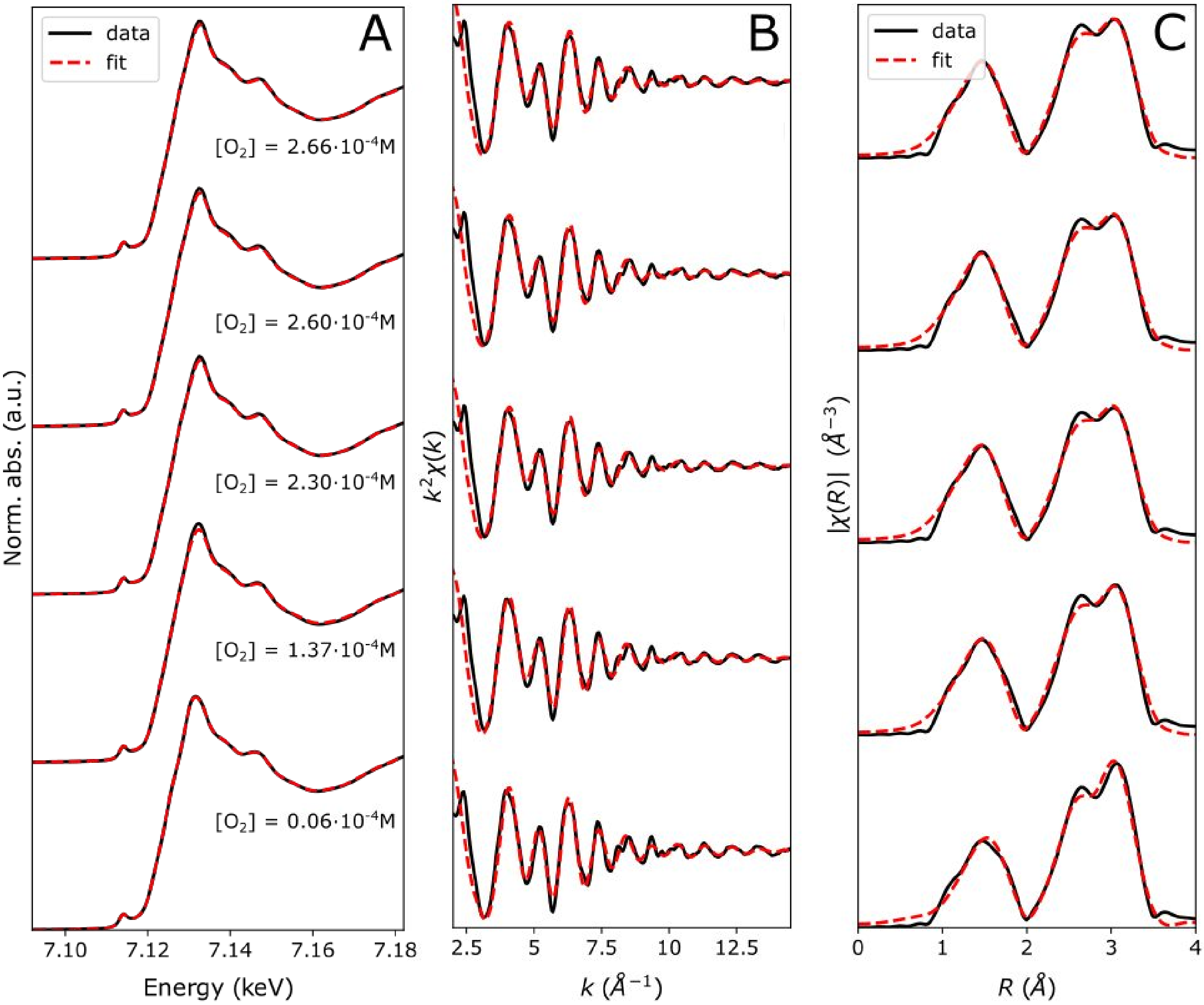

Figure S7: Linear combination fitting analysis of the Fe K-edge (A) XANES and of the least-squares fitting (LSF) analysis on the Fe K-edge EXAFS spectra (B) with the associated EXAFS Fourier-transform (C) for magnetite exposed to ultrapure water at various O_2 concentrations.

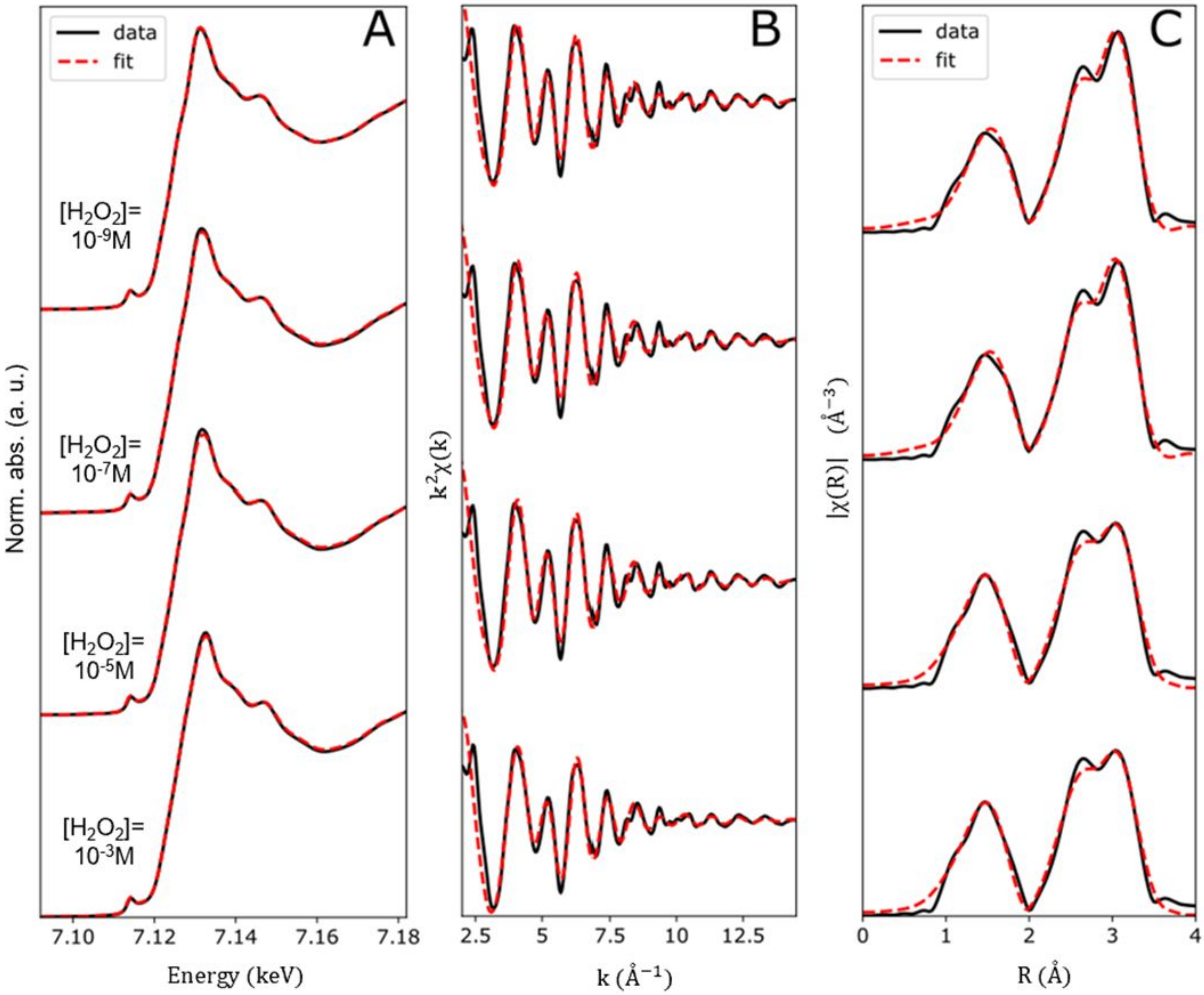

Figure S8: Linear combination fitting analysis of the Fe K-edge (A) XANES and of the least-squares fitting (LSF) analysis on the Fe K-edge EXAFS spectra (B) with the associated EXAFS Fourier-transform (C) of magnetite exposed to ultrapure water at various H_2O_2 concentrations.

ESI Discussion section 2: Detailed findings and structural distortion revealed by Rietveld refinement

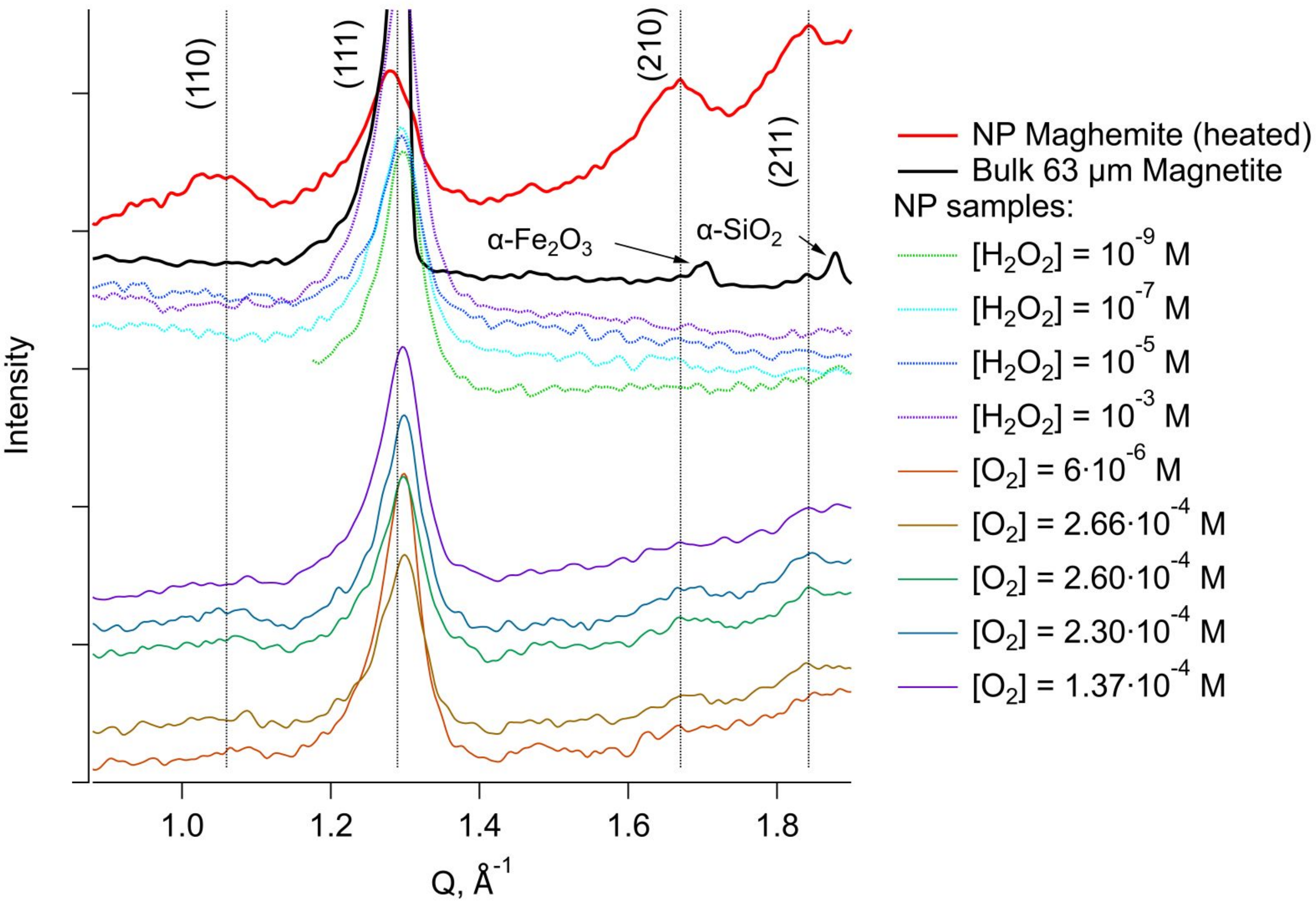

Figure S9. Maghemite superstructure peaks in fully oxidized maghemite endmember, magnetite (superstructure absent, as expected) and partially oxidised samples (observable, but very weak).

As the ESI-Figure 10 shows, fully oxidised maghemite (heated in air) shows well developed peaks as expected from vacancy ordering in $P4_332$ structure onto a single octahedral site. The partially oxidised samples, however, show extremely weak superstructure peaks associated with vacancy ordering, indicating, the samples obtained under mild low temperature oxidative conditions have vacancy distribution very close to random and the development of $P4_332$ structure is slow compared to timescale of the experiment (1 week).

The Ag-XRD was aligned periodically by performing a calibration measurement using a NIST LaB_6 standard covering the entire measurement range of the samples analysed here (starting with $2\theta = 7.7^{\circ}$ as that

is where the first LaB_6 peak is). Si, measured in the same sample geometry (and 0.5mm capillary) was used as an instrument line shape standard. To account for any potential sample displacement, a refinement was performed with the Si sample under assumption it is perfectly crystalline with a lattice parameter of 5.4307 Å. To achieve perfect peak position match, minor correction factors in sample displacement and instrument zero were found. These were then implemented for all sample refinements. Only after such a correction was made, the grain size predicted by XRD closely matched that observed with TEM (TEM initial as synthesized magnetite and maghemite sizes were 10nm for both, while XRD found 10nm for initial as synthesized magnetite and 12nm for maghemite) indicating that this was a correct step.

To assess the suitability of a cubic model for partially oxidised magnetite (e.g., reacting with O_2 or H_2O_2), observed diffraction peaks were each empirically and individually fitted for peak position, intensity and broadness. The fitted peak positions of selected, more intense peaks (all present in the Fd3m structure) were then recorded. The pattern was then indexed looking for an F-cubic unit cells. The peak positions of the found fitted peaks were then recorded, compared to the same (h k l) empirically fitted peaks and the difference was calculated (ESI-Figure 10). In the perfect case there should be no difference between the fitted and the indexed peaks. A significant difference between the two peak positions was, however, observed.

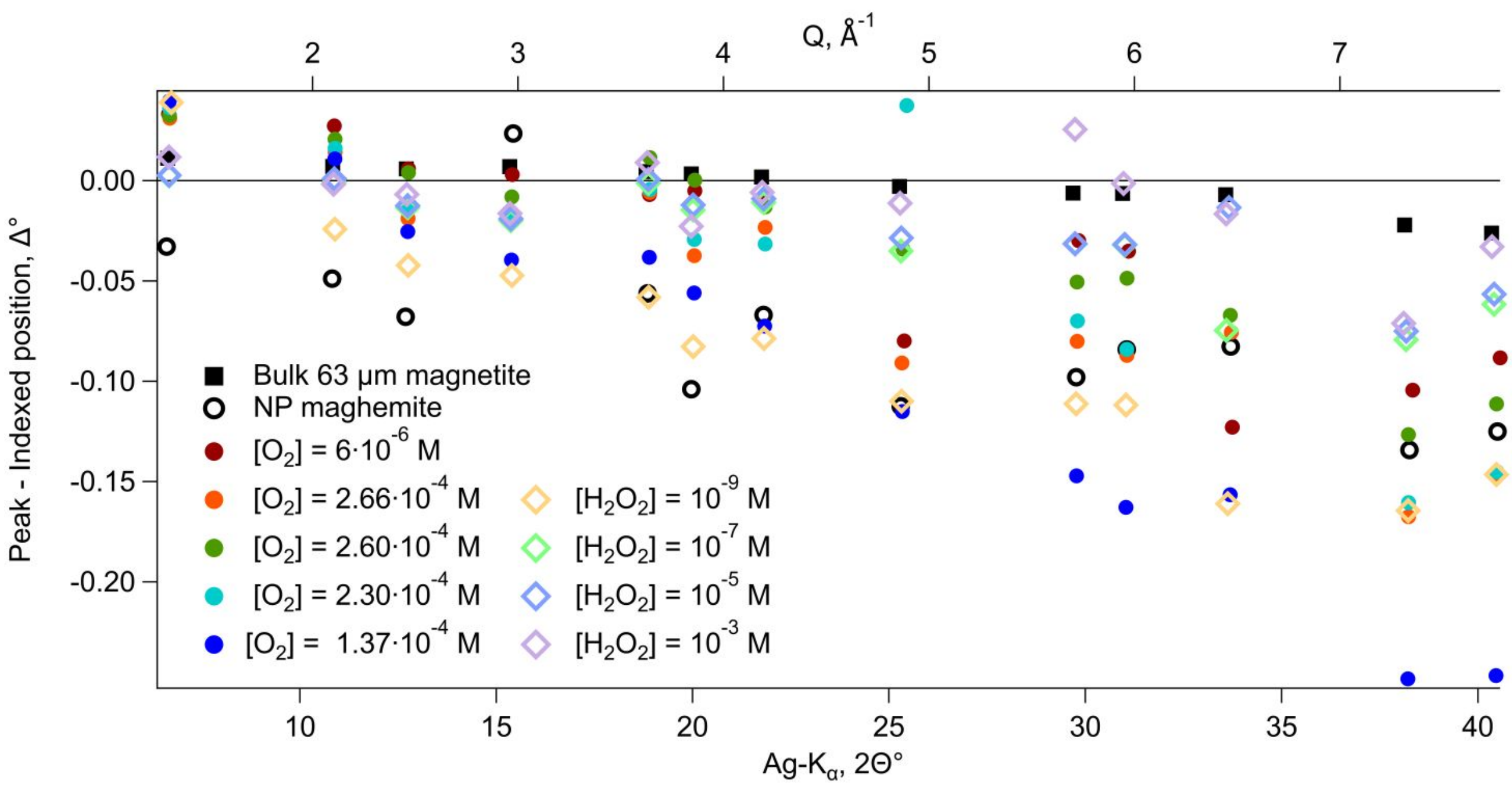

Figure S10. Peak position differences between the actual fitted peak positions and the indexed peak positions considering the collective of the fitted peaks. It can be observed that the more oxidised samples show greater misfit from the expected cubic geometry.

As the results of the peak position analysis show (ESI-Figure 10), bulk cubic magnetite shows fairly minimal deviation from expected cubic peak positions and this deviation could be attributed to sample position error, wobbling or misaligned instrument (even though these were corrected, the error may vary slightly from sample to sample). However, the maximum error observed for bulk magnetite was 0.026° 2θ.

Errors up to almost 10 times, and very commonly 5 times, that were observed and this is too significant error to be dismissed simply due to poor sample alignment when the same sample and instrument alignment procedures were performed.

This leaves structural deviations as the most likely cause. Evidence hinting to this can be found by carful inspection of peak shapes (see ESI-Figure 11, especially 642 peak), where reflections that should be single peaks can have double-peak, shoulder or asymmetric features. Core-shell structure with a diffuse transition between maghemite shell over magnetite core, where, both maghemite and magnetite can have a non-stoichiometric Fe : O ratio, could create similar features. These issues have been highlighted before in (Frison *et al.*, 2013; Cervellino *et al.*, 2014), furthermore, out of all Bragg peaks observed in their scattering patterns 440 peak shows the largest fitting error in refinements, the same as found here (see ESI-Figure 12). This hints at a simple (meaning single phase, unit cell of equivalent size to pure magnetite and not a supercell, simple Lorentz size/strain broadening) cubic model not being able to explain the structure, that could be locally distorted due to the very small size of the nanoparticles and their non-stoichiometric nature or due to wide and complex grain size and stoichiometry distributions.

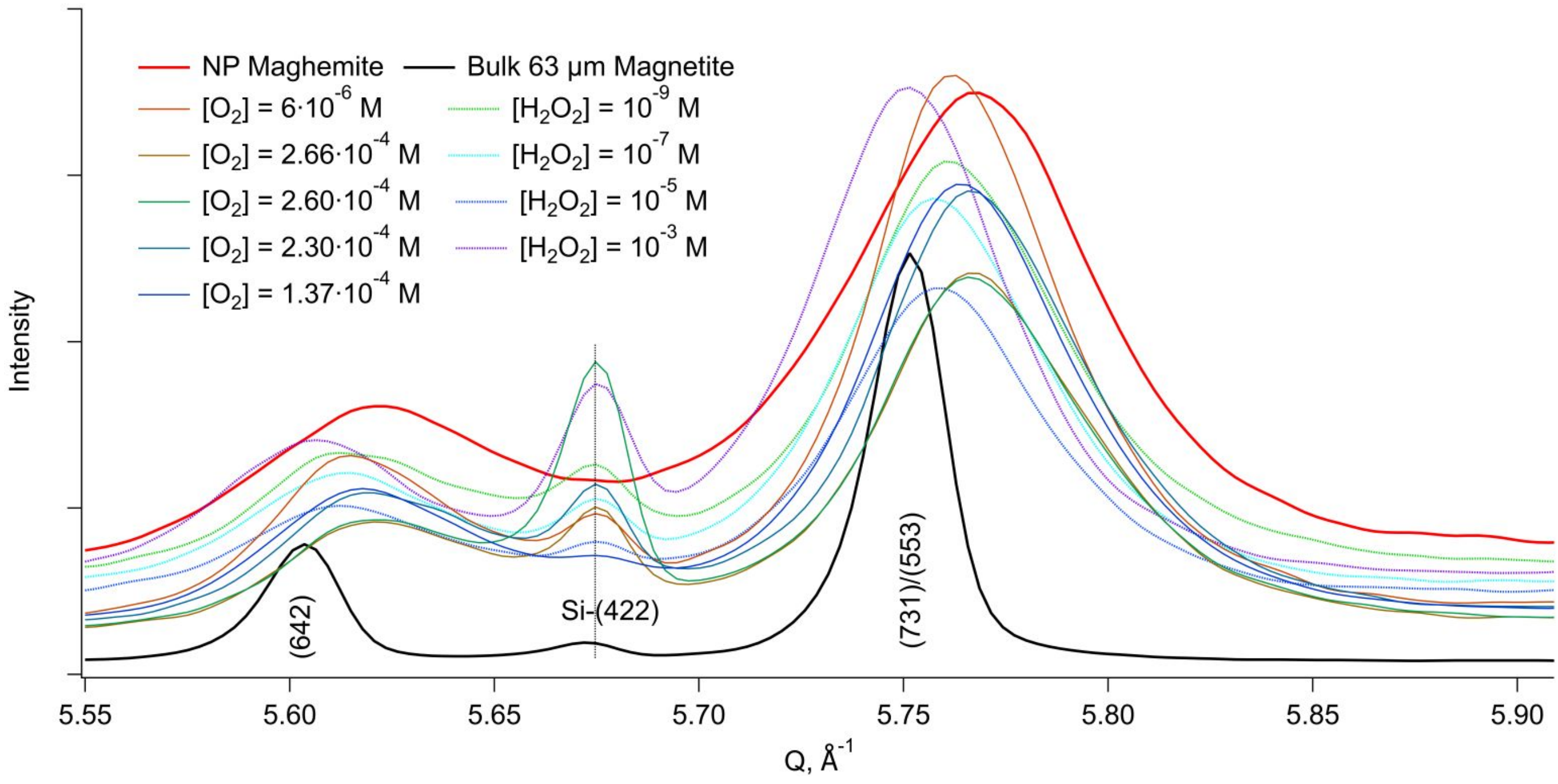

Figure S11. Comparison of peak position and profiles of samples analysed in medium angle range. Si 422 peak is found at the exact same position in all samples, provided statistically significant amount of Si was added to the samples. Multiple samples show complex peak profiles, especially of the 642 peak, suggesting either splitting due to departure from cubic geometry or a complex particle size distribution.

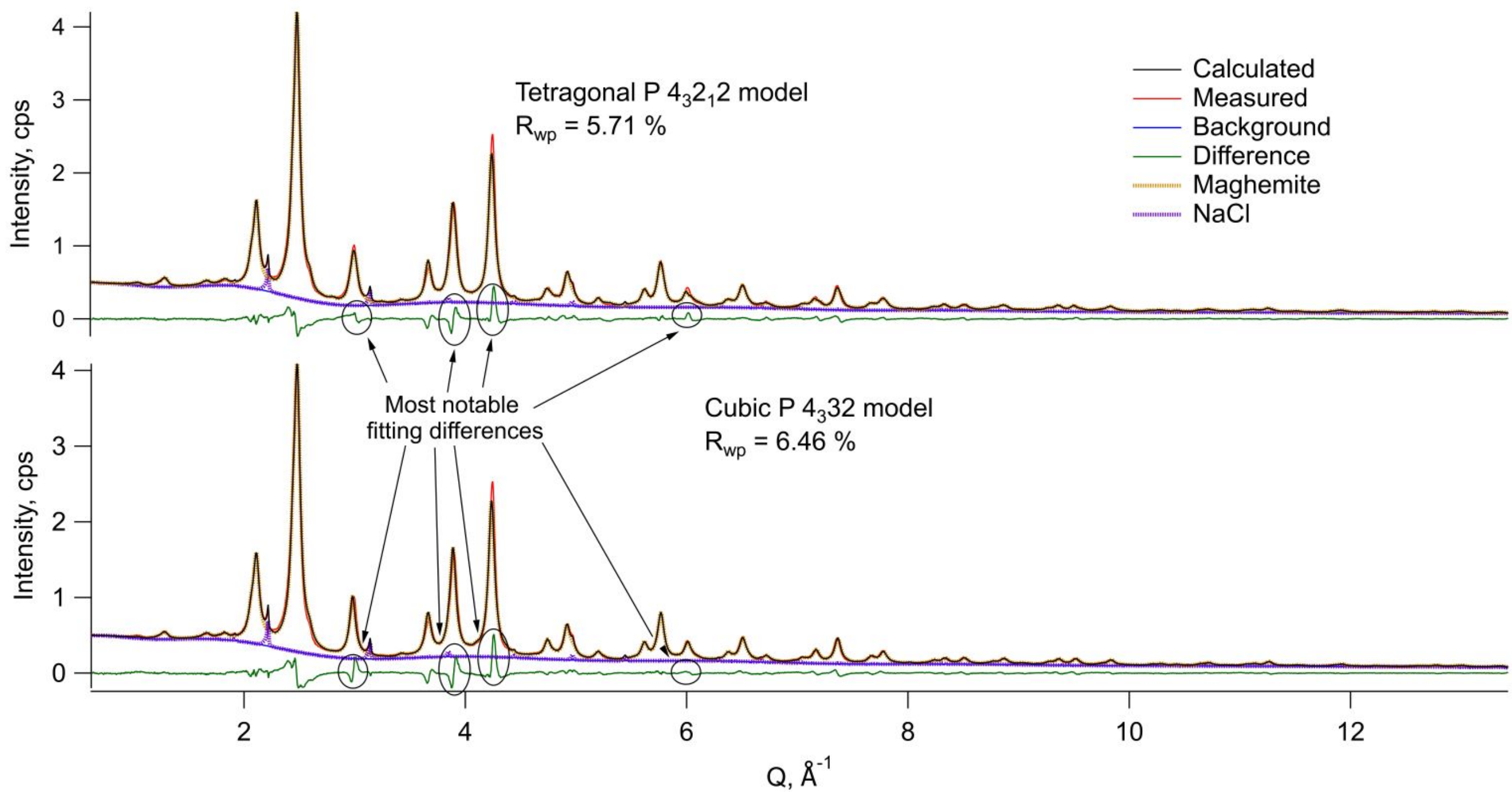

Figure S12. Rietveld refinements of maghemite NPs with a tetragonal $P4_32_12$ model (above) and cubic $P4_332$ model (below). Tetragonal model does show a lower R_{wp} value, however, the reason behind this is unclear as

none of the fundamental errors, such as 440 peak intensity and the other highlighted problems are resolved or significantly improved upon. Therefore, a simpler cubic model was chosen to avoid overfitting.

As discussed in the main text, Fe occupancy did not follow any particular trend, while it is expected for the Fe site occupancy to decrease with increasing conversion of magnetite to maghemite. This is likely occurring due to a strong anticorrelation of Fe occupancy and scale factor. As shown by the lack of clear superstructure peaks in partially oxidized magnetite compared to air heated maghemite (ESI Figure-9), Fe occupancy is similar on all sites, without vacancies forming in particular on a specific site. Therefore, there are no peaks to explain by reducing Fe occupancy of different sites differently. Since the only other atom besides Fe in the structure is relatively very light oxygen, the effect of changing the average Fe occupancy is very similar to changing the scale factor of the phase since the overall pattern intensity will be much more sensitive to Fe amount than peak intensity ratios. This was also observed in the correlation matrix of the refinement where two out of three Fe site occupancies in P4322 maghemite were strongly anticorrelated with pattern scale factor. In addition, since the absorbance was only estimated from sample density and size, differing sample packing densities could have introduced a further complication on this as absorbance is also strongly correlated with scale factor

Bibliographie

1 M. C. Menard, A. C. Marschilok, K. J. Takeuchi and E. S. Takeuchi, *Electrochimica Acta*, 2013, **94**, 320–326.

2 M. Coduri, P. Masala, L. Del Bianco, F. Spizzo, D. Ceresoli, C. Castellano, S. Cappelli, C. Oliva, S. Checchia, M. Allieta, D.-V. Szabo, S. Schlabach, M. Hagelstein, C. Ferrero and M. Scavini, *Nanomaterials*, 2020, **10**, 867.

3 H. Okudera, A. Yoshiasa, K. Murai, M. Okube, T. Takeda and S. Kikkawa, *Journal of Mineralogical and Petrological Sciences*, 2012, **107**, 127–132.

4 F. Maillot, G. Morin, Y. Wang, D. Bonnin, P. Ildefonse, C. Chaneac and G. Calas, *Geochimica et Cosmochimica Acta*, 2011, **75**, 2708–2720.

5 J. Baumgartner, G. Morin, N. Menguy, T. Perez Gonzalez, M. Widdrat, J. Cosmidis and D. Faivre, *Proc. Natl. Acad. Sci. U.S.A.*, 2013, **110**, 14883–14888.

6 A. Corrias, G. Ennas, G. Mountjoy and G. Paschina, *Phys. Chem. Chem. Phys.*, 2000, **2**, 1045–1050.

7 B. Gilbert, J. E. Katz, J. D. Denlinger, Y. Yin, R. Falcone and G. A. Waychunas, *J. Phys. Chem. C*, 2010, **114**, 21994–22001.

8 K. E. Sickafus, L. Minervini, R. W. Grimes, J. A. Valdez, M. Ishimaru, F. Li, K. J. McClellan and T. Hartmann, *Science*, 2000, **289**, 748–751.

9 J.-E. Jørgensen, L. Mosegaard, L. E. Thomsen, T. R. Jensen and J. C. Hanson, *Journal of Solid State Chemistry*, 2007, **180**, 180–185.

10 Z. Somogyvári, E. Sváb, G. Mészáros, K. Krezhov, I. Nedkov, I. Sajó and F. Bourée, *Applied Physics A: Materials Science & Processing*, 2002, **74**, s1077–s1079.

11 S.-S. Lin and M. D. Gurol, *Environ. Sci. Technol.*, 1998, **32**, 1417–1423.
